# NFATc1 Mediates Toll-Like Receptor-Independent Innate Immune Responses during *Trypanosoma cruzi* Infection

**DOI:** 10.1371/journal.ppat.1000514

**Published:** 2009-07-17

**Authors:** Hisako Kayama, Ritsuko Koga, Koji Atarashi, Megumi Okuyama, Taishi Kimura, Tak W. Mak, Satoshi Uematsu, Shizuo Akira, Hiroshi Takayanagi, Kenya Honda, Masahiro Yamamoto, Kiyoshi Takeda

**Affiliations:** 1 Laboratory of Immune Regulation, Department of Microbiology and Immunology, Graduate School of Medicine, Osaka University, Suita, Osaka, Japan; 2 Department of Molecular Genetics, Medical Institute of Bioregulation, Kyushu University, Fukuoka, Japan; 3 WPI Immunology Frontier Research Center, Osaka University, Suita, Osaka, Japan; 4 Ontario Cancer Institute, Princess Margaret Hospital, Toronto, Ontario, Canada, and Department of Medical Biophysics, Advanced Medical Discovery Institute, University of Toronto, Toronto, Ontario Canada; 5 Department of Host Defense, Research Institute for Microbial Diseases, Osaka University, Suita, Osaka, Japan; 6 Department of Cell Signaling, Graduate School, Tokyo Medical and Dental University, Tokyo, Japan; University of Wisconsin-Madison, United States of America

## Abstract

Host defense against the intracellular protozoan parasite *Trypanosoma cruzi* depends on Toll-like receptor (TLR)-dependent innate immune responses. Recent studies also suggest the presence of TLR-independent responses to several microorganisms, such as viruses, bacteria, and fungi. However, the TLR-independent responses to protozoa remain unclear. Here, we demonstrate a novel TLR-independent innate response pathway to *T. cruzi*. *Myd88* ^−/−^ *Trif* ^−/−^ mice lacking TLR signaling showed normal *T. cruzi*-induced Th1 responses and maturation of dendritic cells (DCs), despite high sensitivity to the infection. IFN-γ was normally induced in *T. cruzi*-infected *Myd88* ^−/−^ *Trif* ^−/−^ innate immune cells, and further was responsible for the TLR-independent Th1 responses and DC maturation after *T. cruzi* infection. *T. cruzi* infection induced elevation of the intracellular Ca^2+^ level. Furthermore, *T. cruzi*-induced IFN-γ expression was blocked by inhibition of Ca^2+^ signaling. NFATc1, which plays a pivotal role in Ca^2+^ signaling in lymphocytes, was activated in *T. cruzi*-infected *Myd88^−/−^Trif^−/−^* innate immune cells. *T. cruzi*-infected *Nfatc1* ^−/−^ fetal liver DCs were impaired in IFN-γ production and DC maturation. These results demonstrate that NFATc1 mediates TLR-independent innate immune responses in *T. cruzi* infection.

## Author Summary

*Trypanosoma cruzi* is an intracellular protozoan parasite that causes Chagas diseases in humans. Invasion of *T. cruzi* into the host is sensed by Toll-like receptors (TLRs), which recognize microbial components that are present in microbes but not in the host. TLRs are essential for the initiation of immune responses against pathogens. Recent evidence indicates the presence of TLR-independent mechanisms for the recognition of microbes, such as bacteria, viruses, and fungi. However, TLR-independent recognition of protozoa remains unknown. We found that immune responses against *T. cruzi* were induced even in the absence of TLR signaling. The TLR-independent responses were found to be mediated by IFN-γ production in innate immune cells. Furthermore, the TLR-independent IFN-γ production was revealed to be mediated by Ca^2+^-dependent activation of NFATc1, which has been shown to play a pivotal role in cytokine production in T lymphocytes. Our study provides a novel mechanism for the TLR-independent innate immune response against protozoan parasites. It is also worth noting that the host defense mechanism utilizes a factor (Ca^2+^) that is a prerequisite for the survival of intracellular protozoan parasites.

## Introduction

The host defense against invasion of intracellular pathogens relies on Th1 cell-derived IFN-γ that activates macrophages to kill the engulfed pathogens [1]. Toll-like receptor (TLR)-mediated recognition of pathogens has been established to induce activation of innate immune cells such as dendritic cells (DCs) and subsequent development of Th1 cells [2],[3]. However, recent evidence also indicates the presence of TLR-independent mechanisms for the recognition of microorganisms such as bacteria, viruses, and fungi [4],[5]. Accordingly, TLR-independent mechanisms for Th1 development have been demonstrated in several infectious models such as fungal and bacterial infections [6],[7]. However, TLR-independent recognition of protozoa remains unknown.

*Trypanosoma cruzi* is an intracellular protozoan parasite that causes Chagas' disease, a chronic disorder characterized by cardiomyopathy and malformation of the intestine [8]. Several components of *T. cruzi* have been shown to induce TLR-dependent activation of innate immunity and subsequent development of Th1 cells [9]–[14]. The absence of TLR-dependent activation of innate immunity results in high susceptibility to *T. cruzi* infection [15],[16] due to defective type I interferon (IFN)-mediated induction of the GTPase IFN-inducible p47 (IRG47) [17]. Invasion of infective metacyclic trypomastigotes of *T. cruzi* into host cells induces a close interaction between the parasites and the host, because *T. cruzi* utilize several host-derived factors in order to establish the infection. These include activation of Ca^2+^ signaling pathways and phosphatidylinositol-3 kinases [18]–[20]. However, it remains unclear how *T. cruzi*-mediated activation of host cytoplasmic signaling pathways is regulated and whether it is TLR-dependent or -independent.

In T cells, the nuclear factor of activated T cells (NFAT) family of transcription factors has been shown to mediate production of cytokines including IFN-γ [21],[22]. The NFAT family of proteins comprises four closely related members (NFATc1, NFATc2, NFATc3, and NFATc4) that are activated by Ca^2+^ signaling, and NFAT5 that is regulated by osmotic stress. The role of NFAT proteins in T cells has been well characterized [21],[22]. However, little is known about the role of NFAT proteins in innate immune responses, although some of the NFAT members are highly expressed and can modulate gene induction in macrophages (Mφ) [23],[24].

Here, we analyzed the mechanisms of TLR-independent activation of innate immunity during *T. cruzi* infection using *T. cruzi*-infected *Myd88^−/−^Trif^−/−^* mice. Our results demonstrate that NFATc1 mediates TLR-independent induction of IFN-γ in innate immune cells, leading to effective Th1 responses during *T. cruzi* infection.

## Results

### Normal Th1 response in *T. cruzi*-infected *Myd88* ^−/−^ *Trif* ^−/−^ mice

**Figure 1:**
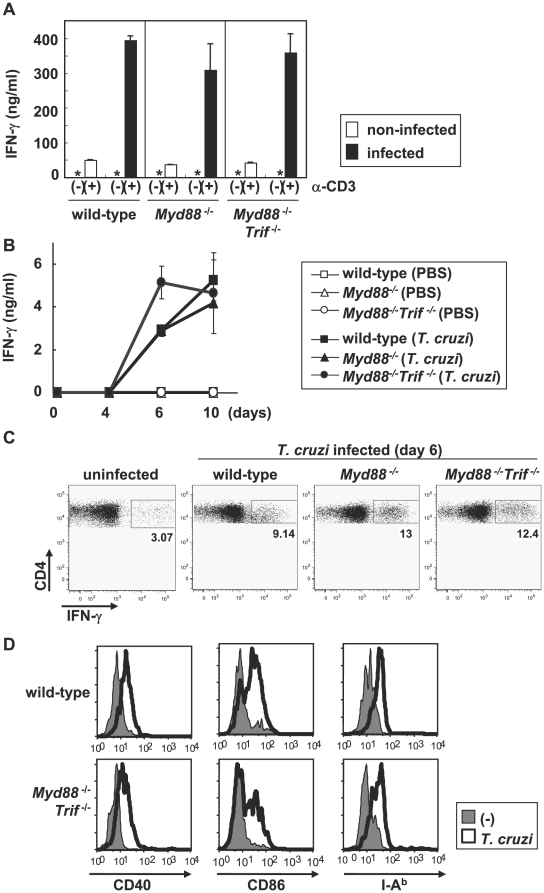
Th1 response and DC maturation in *T. cruzi*-infected *Myd88* ^−/−^ *Trif* ^−/−^ mice. (A, B) Splenic CD4^+^ T cells were isolated from wild-type, *Myd88* ^−/−^ and *Myd88* ^−/−^ *Trif* ^−/−^ mice after 6 days (A) or the indicated days (B) of *T. cruzi* infection or PBS injection, and stimulated with anti-CD3 antibody (A) or freeze-thawed *T. cruzi* in the presence of antigen presenting cells (B). After 24 h, supernatants were assayed for IFN-γ production by ELISA. Data are mean+s.d. of triplicate determination and a representative result of at least three independent experiments. In each experiment, two or three mice in each group were used. *: not detected. (C) Splenocytes were isolated from *T. cruzi*-infected or none-infected wild-type, *Myd88^−/−^* and *Myd88^−/−^Trif^−/−^* mice, and stimulated with 1 µg/ml ionomycin plus 50 ng/ml PMA. After surface staining with APC-conjugated anti-CD4 Ab, the cells were permeabilized and then stained with PE-conjugated anti-IFN-γ Ab, and analyzed by flow cytometry. The percentage of IFN-γ-producing CD4^+^ cells of individual mice is shown. (D)*T. cruzi*-infected or none infected bone marrow DCs of wild-type and *Myd88* ^−/−^ *Trif* ^−/−^ were stained for expression of CD40, CD86, and I-A^b^, and then analyzed by FACS.

Previously, we demonstrated that mice lacking both MyD88 and TRIF, in which TLR-dependent activation was abolished, are highly sensitive to infection with *T. cruzi* [17]. Because TLRs have been shown to control development of Th1 cells, we analyzed Th1 responses in *T. cruzi*-infected mice. Mice were intraperitoneally (i.p.) infected with *T. cruzi* trypomastigotes, and at 6 days of infection CD4^+^ T cells were isolated from the spleen and stimulated with anti-CD3 antibody (Ab) (Figure 1A). In *T. cruzi*-infected wild-type mice, there was considerable production of IFN-γ compared with that in non-infected control mice, indicating induction of potent Th1 responses. In *Myd88* ^−/−^ and *Myd88* ^−/−^ *Trif* ^−/−^ mice, IFN-γ production was similar to that in wild-type mice following *T. cruzi* infection. Next, we analyzed the antigen-specific Th1 response at 0, 4, 6, and 10 days after *T. cruzi* infection by stimulating CD4^+^ T cells with freeze-thawed *T. cruzi* in the presence of antigen presenting cells (APC) (Figure 1B). This stimulation induced marked production of IFN-γ at 6 and 10 days of the infection in wild-type mice. Even in CD4^+^ T cells derived from *T. cruzi*-infected *Myd88* ^−/−^ and *Myd88* ^−/−^ *Trif* ^−/−^ mice, antigen-specific production of IFN-γ was induced to levels similar to that of wild-type mice. Thus, the antigen-specific Th1 response was not impaired in *Myd88* ^−/−^ and *Myd88* ^−/−^ *Trif* ^−/−^ mice. We also analyzed IFN-γ production from CD4^+^ T cells by intracellular staining (Figure 1C, Figure S1A). The number of IFN-γ-producing CD4^+^ T cells was almost equally elevated in wild-type, *Myd88* ^−/−^ and *Myd88* ^−/−^ *Trif* ^−/−^ mice at 6 days (Figure 1C) as well as at 10 days (Figure S1A) after infection. Consistent with previous studies [25],[26], the number of IFN-γ producing CD8^+^ T cells and NK1.1^+^ natural killer cells was not increased at 10 days after *T. cruzi* infection (Figure S1B).

Development of Th1 cells is critically controlled by DCs [27],[28]. In addition, stimulation of TLRs induces maturation of DCs [3]. Therefore, we analyzed expression of MHC class II and co-stimulatory molecules on *T. cruzi*-infected DCs. Bone marrow-derived DCs (BMDCs) were infected with *T. cruzi* trypomastigotes for 6 h, then were washed, cultured for 48 h, and analyzed for expression of MHC class II, CD40, and CD86 by flow cytometry (Figure 1D). *T. cruzi* infection resulted in enhanced expression of these molecules in wild-type BMDCs. Expression was also increased in BMDCs derived from *Myd88* ^−/−^ *Trif* ^−/−^ mice after *T. cruzi* infection, indicating normal maturation of *T. cruzi*-infected DCs of *Myd88* ^−/−^ *Trif* ^−/−^ mice. Thus, Th1 cell development and DC maturation were induced during *T. cruzi* infection even in the absence of TLR-dependent activation of innate immunity.

### IFN-γ induction in *T. cruzi*-infected *Myd88* ^−/−^ *Trif* ^−/−^ DCs and macrophages

**Figure 2:**
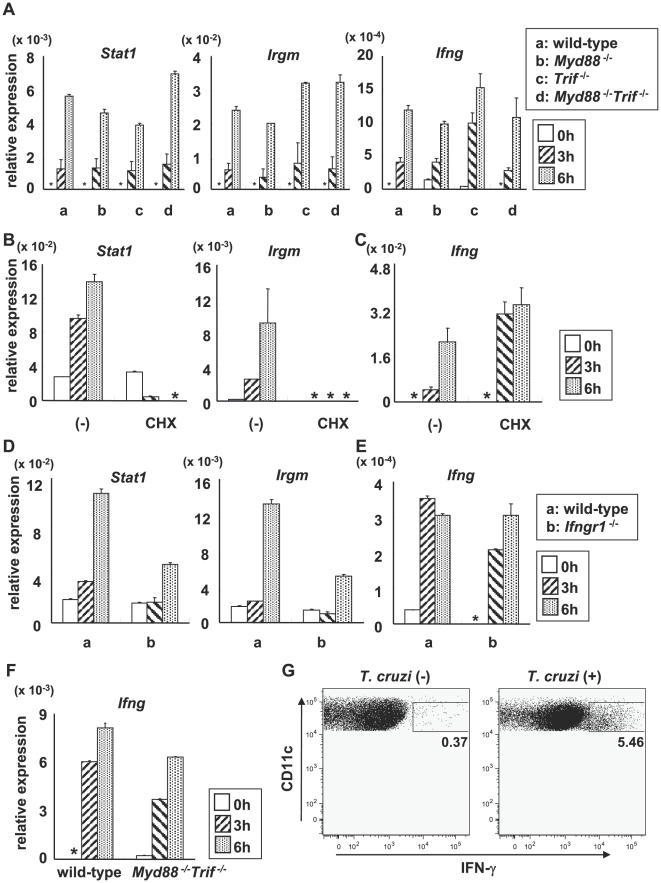
Expression of IFN-γ-inducible genes in *T. cruzi*-infected *Myd88^−/−^Trif^−/−^* DCs. (A) Bone marrow DCs from wild-type, *Myd88* ^−/−^, *Trif* ^−/−^, and *Myd88* ^−/−^ *Trif* ^−/−^ mice were infected with *T. cruzi* for 3 or 6 h, and total RNA was isolated and mRNA expression of *Stat1*, *Irgm* and *Ifng* was quantified by real-time RT-PCR and normalized to the level of elongation factor-1α (EF1α). *: not detected (B, C) Bone marrow DCs from wild-type mice were pretreated with 1 µg/ml cycloheximide (CHX) for 5 min, then infected with *T. cruzi* for the indicated periods. Next, mRNA expression of *Stat1*, *Irgm* (B) and *Ifng* (C) was analyzed by real-time RT-PCR. *: not detected. Data are representative of three independent experiments. (D, E) Bone marrow DCs from wild-type and *Ifngr1* ^−/−^ mice were infected with *T. cruzi* for 3 or 6 h. Next, mRNA expression of *Stat1*, *Irgm* (D) and *Ifng* (E) was analyzed. *: not detected (F) CD11c^high^B220^−^NK1.1^−^ cells were isolated from the spleen of wild-type and *Myd88^−/−^Trif^−/−^* mice, and then *T. cruzi*-induced expression of *Ifng* was analyzed. Data are mean+s.d. of triplicate determination and a representative result of at least three independent experiments. (G) Splenocytes from wild-type mice were infected with *T. cruzi* for 18 h. After surface staining with APC-conjugated anti-CD11c Ab, the cells were permeabilized and then stained with PE-conjugated anti-IFN-γ Ab, and analyzed by flow cytometry. Representative results are shown from four independent experiments. The percentage of IFN-γ-producing CD11c^+^ cells of individual mice is shown.

*T. cruzi* infection induced maturation of DCs in the absence of TLR signaling. Therefore, we screened genes that were normally induced in *T. cruzi*-infected DCs of *Myd88* ^−/−^ *Trif* ^−/−^ mice. BMDCs from wild-type, *Myd88* ^−/−^ and *Myd88* ^−/−^ *Trif* ^−/−^ mice were infected with *T. cruzi* trypomastigotes for 6 h, then mRNA was extracted and used for DNA microarray analysis. Approximately 80% of genes that were induced in *T. cruzi*-infected wild-type DCs (about 4000 genes) were MyD88-dependent, as the *T. cruzi*-mediated induction was reduced in *Myd88* ^−/−^ DCs (Figure S2). Some of the genes that were normally induced in *Myd88* ^−/−^ DCs, but not induced in *Myd88* ^−/−^ *Trif* ^−/−^ DCs (MyD88/TRIF-dependent genes; 14% of genes that were induced in wild-type DCs) are known to be induced by type I IFNs. In addition, a majority of the small number of genes that were induced even in *Myd88* ^−/−^ *Trif* ^−/−^ DCs (6%) were IFN-γ-inducible genes (Figure S3). In order to corroborate that IFN-γ-inducible genes are normally induced in *T. cruzi*-infected *Myd88* ^−/−^ *Trif* ^−/−^ DCs, we analyzed mRNA expression of *Ifng* and IFN-γ-inducible genes, including *Stat1*, and *Irgm*, by real-time RT-PCR (Figure 2A). *T. cruzi* infection resulted in robust induction of *Ifng*, *Stat1*, and *Irgm* in wild-type, *Myd88* ^−/−^, *Trif* ^−/−^, and *Myd88* ^−/−^ *Trif* ^−/−^ DCs. *T. cruzi*-induced expression of *Stat1* and *Irgm* in wild-type DCs was inhibited by addition of a de novo protein synthesis inhibitor, cycloheximide (CHX) (Figure 2B). In contrast, CHX did not inhibit *T. cruzi*-induced *Ifng* expression (Figure 2C). Next, in order to analyze whether the expression of the IFN-γ-inducible genes was secondary to induction of *Ifng*, we used BMDCs derived from *Ifngr1* ^−/−^ mice in which the IFN-γ-mediated response was abolished (Figure 2D, E). In *Ifngr1* ^−/−^ BMDCs, *T. cruzi*-mediated induction of *Stat1* and *Irgm* was reduced, whereas induction of *Ifng* was unimpaired. These data indicate that *Ifng* was induced primarily in response to *T. cruzi* infection, and *Stat1* and *Irgm* were induced secondary to *Ifng* induction. In peritoneal Mφ, similar patterns of *T. cruzi*-mediated gene expression were observed (Figure S4). Recently, the CD11c^low^B220^+^NK1.1^+^ subset of cells was identified as a natural killer (NK) cell subset with a high capacity for IFN-γ production in response to IL-12 or a TLR9 ligand [29]–[31]. To exclude the possibility of contamination of these cells in preparation of BMDCs or Mφ, we purified CD11c^high^B220^−^NK1.1^−^ population from the spleen, and analyzed for IFN-γ expression (Figure S5A). CD11c^high^B220^−^NK1.1^−^ cells showed very low levels of IFN-γ expression in response to IL-12/IL-18 stimulation compared with the NK cell subset (Figure S5B). However, these cells from wild-type and *Myd88^−/−^Trif^−/−^* mice expressed IFN-γ in response to *T. cruzi* infection (Figure 2F). Flow cytometric analysis further demonstrated that *T. cruzi*-infected CD11c^high^ splenic DCs expressed IFN-γ protein (Figure 2G). These findings indicate that *T. cruzi* infection induces IFN-γ production in DCs.

### IFN-γ-mediated DC maturation and Th1 responses in *T. cruzi*-infected *Myd88* ^−/−^ *Trif* ^−/−^ mice

**Figure 3:**
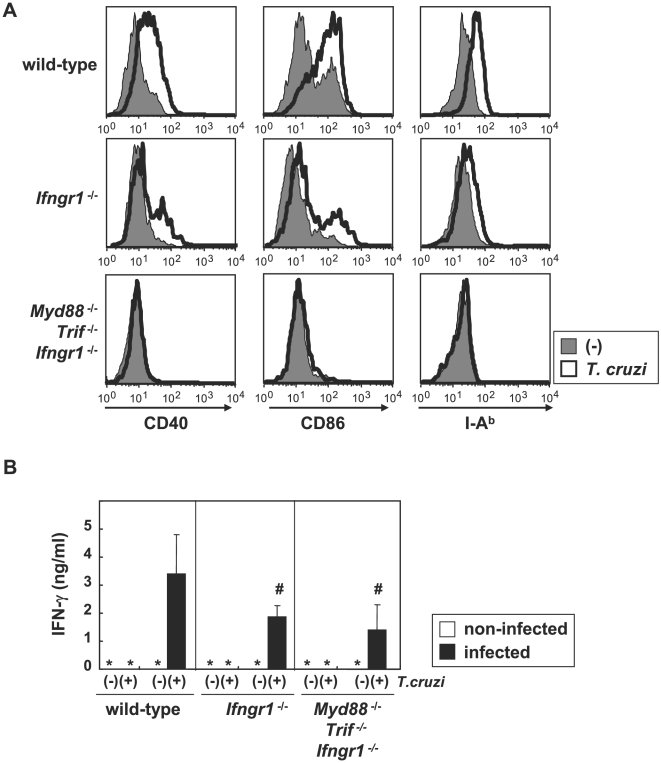
IFN-γ dependent DC maturation and Th1 response in *T. cruzi* infection. (A) Bone marrow DCs of wild-type, *Ifngr1* ^−/−^, and *Myd88* ^−/−^ *Trif* ^−/−^ *Ifngr1* ^−/−^ mice were infected with *T. cruzi* for 6 h, then washed and cultured for 48 h. The cells were analyzed for expression of CD40, CD86, and I-A^b^ using flow cytometry. Representative results are shown from three independent experiments. (B) Wild-type, *Ifngr1* ^−/−^, and *Myd88* ^−/−^ *Trif* ^−/−^ *Ifngr1* ^−/−^ mice were infected with *T. cruzi*. At six days after infection, CD4^+^ T cells were isolated from the spleen, and then stimulated with freeze-thawed *T. cruzi* in the presence of antigen presenting cells. After 24 h, supernatants were collected and assayed for IFN-γ production by ELISA. The values are the means+s.d. of four independent experiments each carried out in triplicate. #:*P*<0.05.

Next, we analyzed whether IFN-γ was involved in TLR-independent DC maturation and Th1 responses during *T. cruzi* infection. In BMDCs derived from *Ifngr1* ^−/−^ mice, *T. cruzi*-induced enhancement of CD40, CD86, and MHC class II was partially reduced (Figure 3A). Furthermore, expression of these molecules was completely abolished in *T. cruzi*-infected DCs of *Myd88* ^−/−^ *Trif* ^−/−^ *Ifngr1* ^−/−^ mice. Enhanced expression of these molecules in response to exogenous IFN-γ was not observed in *Ifngr1* ^−/−^ BMDCs (Figure S6A). These findings indicate that IFN-γ produced from *T. cruzi*-infected DCs mediated DC maturation. We also analyzed IFN-γ production from splenic CD4^+^ T cells of *T. cruzi*-infected mice (Figure 3B). In both *Ifngr1* ^−/−^ and *Myd88* ^−/−^ *Trif* ^−/−^ *Ifngr1* ^−/−^ mice, *T. cruzi* antigen-dependent production of IFN-γ was severely reduced. Importance of IFN-γ production was further underscored by the finding that *Ifngr1* ^−/−^ mice were more sensitive to *T. cruzi* infection than *Myd88* ^−/−^ *Trif* ^−/−^ mice (Figure S6B). These results demonstrate that IFN-γ mediates TLR-independent DC maturation and Th1 development during *T. cruzi* infection.

Importance of IL-12 in Th1 cell development has been established [32]. Indeed, IL-12p40-deficient mice were highly susceptible to *T. cruzi* infection with severely reduced Th1 responses ([33],[34] and Figure S7A). In addition, IL-12p40 concentration in the serum was decreased in *T. cruzi*-infected *Ifngr1^−/−^* mice (Figure S7B). In *T. cruzi*-infected *Myd88* ^−/−^ *Trif* ^−/−^ mice, IL-12p40 production was severely reduced, but still induced [17], suggesting that IL-12 is produced via TLR-dependent and -independent pathways. Thus, IFN-γ, which is produced via the TLR-independent pathways, might induce IL-12p40 to activate T cells to fully differentiate into Th1 cells.

### Involvement of Ca^2+^ signaling in IFN-γ induction in *T. cruzi*-infected *Myd88* ^−/−^ *Trif* ^−/−^ cells

**Figure 4:**
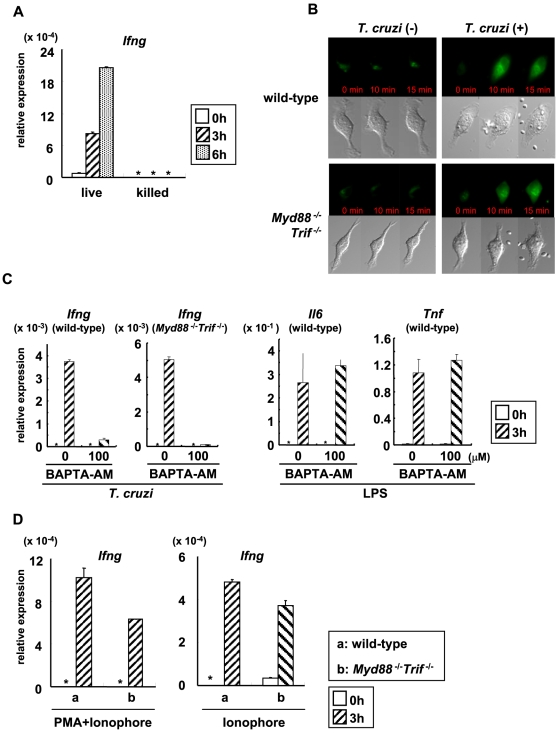
Ca^2+^ signaling-dependent IFN-γ production in *T. cruzi*-infected DCs. (A) Bone marrow DCs were stimulated with live *T. cruzi* or *T. cruzi* killed by repeated freeze-thaw steps for the indicated periods. Next, total RNA was isolated and analyzed for *Ifng* expression by real-time RT-PCR. The fold differences of each sample relative to EF1α are shown. *: not detected. (B) Bone marrow Mφ from wild-type and *Myd88^−/−^Trif^−/−^* mice were treated with Fluo-4AM for 30 min, and washed. Then, cells which were infected or non-infected with *T. cruzi* were analyzed by fluorescence microscopy at the indicated periods. Representative of three independent experiments. (C) Bone marrow DCs from wild-type and *Myd88* ^−/−^ *Trif* ^−/−^ mice were pre-treated with BAPTA-AM (100 µM) for 30 min, and washed. Then, cells were infected with *T. cruzi* for 3 h. Expression of *Ifng* was analyzed by real-time RT PCR. Bone marrow DCs from wild-type mice were stimulated with LPS (100 ng/ml) for 3 h, and analyzed for expression of *Il6* and *Tnf*. (D) Bone marrow Mφ from wild-type and *Myd88^−/−^ Trif^−/−^* mice were stimulated with 5 µM Ca^2+^ ionophore plus 50 ng/ml PMA or 5 µM Ca^2+^ ionophore for the indicated periods, and analyzed for expression of *Ifng*. Data are mean+s.d., and representative one of three independent experiments. *: not detected.

Next, we analyzed the molecular mechanisms for TLR-independent induction of IFN-γ after *T. cruzi* infection. In *Myd88* ^−/−^ *Trif* ^−/−^ DCs, *T. cruzi*-induced phosphorylation of MAP kinases such as ERK, p38, and JNK, as well as degradation of IκBα was not observed at all (Figure S8A). In addition, *T. cruzi* infection did not induce DNA binding activity of NF-κB in *Myd88* ^−/−^ *Trif* ^−/−^ DCs (Figure S8B). Thus, *T. cruzi*-mediated activation of NF-κB and MAP kinases was not induced in the absence of TLR signaling. Next, we stimulated DCs with *T. cruzi* trypomastigotes killed by repeated freeze-thaw steps. Live *T. cruzi*, but not killed parasites, induced *Ifng* expression (Figure 4A). Because many studies have demonstrated that *T. cruzi* utilize the host Ca^2+^ signaling to establish the infection [35], we assessed the intracellular Ca^2+^ concentration in *T. cruzi*-infected BMMφ using a fluorescent Ca^2+^ indicator Fluo-4 AM (Figure 4B, Figure S9A, B). *T. cruzi* infection led to rapid increase in intracellular Ca^2+^ level in both wild-type and *Myd88^−/−^Trif^−/−^* Mφ, which returned to the basal level after 18 min of the infection. Epimastigotes, which are not able to invade the host cells, did not induce the elevation of Ca^2+^ concentration in Mφ (Figure S9C). These results prompted us to examine whether Ca^2+^ mobilization induced by intracellular invasion of *T. cruzi* contributed to the TLR-independent *Ifng* induction. Accordingly, we treated wild-type and *Myd88* ^−/−^ *Trif* ^−/−^ BMDCs with an intracellular Ca^2+^ chelator, bis-(o-aminophenoxy)-ethane-N,N,N′,N′-tetraacetic acid tetra (acetoxymethyl) ester (BAPTA-AM), and infected with *T. cruzi*. In BAPTA-AM pre-treated DCs, *T. cruzi*-induced *Ifng* expression was severely reduced, although lipopolysaccharide (LPS)-induced response was not impaired (Figure 4C). In this condition, *T. cruzi*-induced elevation of intracellular Ca^2+^ concentration was severely reduced (Figure S10). In addition, stimulation with both phorbol myristate acetate (PMA)/Ca^2+^ ionophore or Ca^2+^ ionophore alone, which mimics Ca^2+^ signaling, induced expression of *Ifng* in both wild-type and *Myd88^−/−^Trif^−/−^* Mφ (Figure 4D). Taken together, these findings indicate that *T. cruzi*-dependent intracellular Ca^2+^ mobilization mediates TLR-independent *Ifng* induction.

### NFATc1 activation in *T. cruzi*-infected *Myd88* ^−/−^ *Trif* ^−/−^ cells

**Figure 5:**
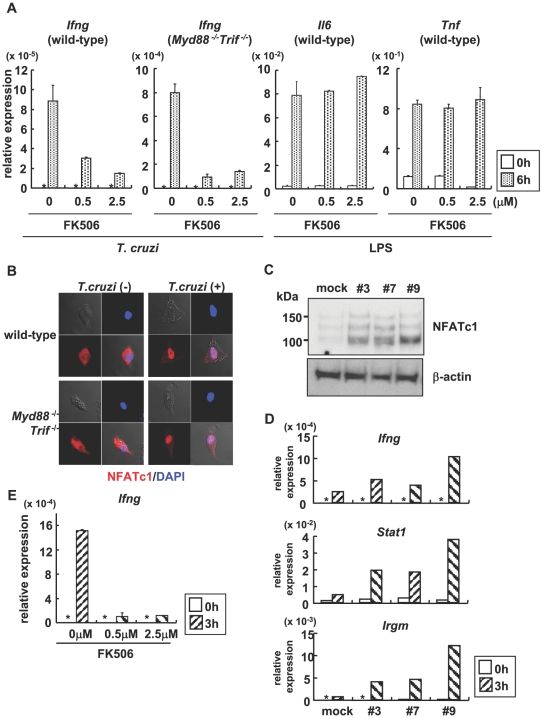
*T. cruzi*-induced activation of NFATc1 in *Myd88* ^−/−^ *Trif* ^−/−^ DCs and Mφ. (A) Wild-type and *Myd88* ^−/−^ *Trif* ^−/−^ bone marrow Mφ were infected with *T. cruzi* or stimulated with LPS for 6 h in the presence or absence of FK506 (0.5 µM or 2.5 µM). Next, total RNA was isolated and analyzed for *Ifng*, *Tnf* or *Il6* expression by real-time RT-PCR. The fold differences of each sample relative to EF1α are shown. *: not detected. (B) Bone marrow Mφ from wild-type and *Myd88* ^−/−^ *Trif* ^−/−^ mice were transfected with the NFATc1 expression plasmid. Cells were then infected with *T. cruzi* for 30 min., and stained with anti-NFATc1 antibody (red) and DAPI (blue). (C) RAW 264.7 cell clones (designated #3, #7, and #9) transfected with the NFATc1 expression plasmid were analyzed for expression of NFATc1 by immunoblot with antibodies specific for NFATc1 and β-actin. (D) RAW 264.7 cells expressing NFATc1 were infected with *T. cruzi* for 3 h. Next, total RNA was extracted and used for real-time RT-PCR analysis using primers specific for *Ifng*, *Stat1*, and *Irgm*. (E) RAW 264.7 cells expressing NFATc1 (clone #9) were infected with *T. cruzi* for 3 h in the presence or absence of FK506, and total RNA was extracted. Real-time RT-PCR analysis was performed using primers specific for *Ifng*. *: not detected. Data are mean+s.d., and a representative result of at least three independent experiments.

In the host cells, especially in T lymphocytes, Ca^2+^ mobilization induces activation of cytokine genes via calmodulin/calcineurin-dependent activation of the transcription factor NFAT. Therefore, we treated *Myd88* ^−/−^ *Trif* ^−/−^ Mφ with FK506 to block calcineurin activation, and infected with *T. cruzi*. Treatment of FK506 resulted in a marked decrease in *T. cruzi*-induced expression of *Ifng*, despite normal LPS-induced response (Figure 5A). Among NFAT members, *Nfatc1*, *Nfatc3*, and *Nfat5* mRNA were abundantly expressed in BMDCs (Figure S11). A previous study has shown that NFATc1 increased anti-CD3/anti-CD28-induced IFN-γ promoter activity in T cells [36]. Furthermore, it has been demonstrated that IFN-γ production was normal in NFATc3-deficient splenocytes [37]. In addition, NFAT5 has been shown to be activated by osmotic stress, but not by Ca^2+^ signaling [38]. Thus, we focused on NFATc1. In wild-type and *Myd88* ^−/−^ *Trif* ^−/−^ BMMφ, *T. cruzi* trypomastigotes infection induced nuclear translocation of NFATc1 (Figure 5B). *T. cruzi*-induced nuclear translocation of NFATc1 was blocked by the pre-treatment with BAPTA-AM in wild-type and *Myd88* ^−/−^ *Trif* ^−/−^ BMMφ (Figure S12A, B). These results indicate that NFATc1 is activated in response to *T. cruzi* infection in a TLR-independent manner. Next, we analyzed whether NFATc1 was involved in the *T. cruzi*-induced IFN-γ production. We obtained RAW264.7 macrophage clones expressing different levels of NFATc1 (Figure 5C). In NFATc1 expressing RAW264.7 cells, *T. cruzi*-induced expression of *Ifng*, *Stat1*, and *Irgm* was enhanced, and the extent of fold-induction correlated with the NFATc1 expression level (Figure 5D). *T. cruzi*-induced *Ifng* expression was severely reduced in the presence of FK506 (Figure 5E). These findings indicate the possible involvement of NFATc1 in mediating IFN-γ production in *T. cruzi*-infected innate immune cells.

**Figure 6:**
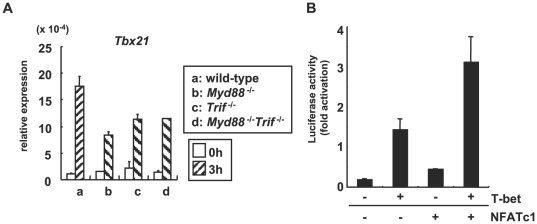
NFATc1 and T-bet had a synergistic effect on activation of the *Ifng* promoter. (A) Peritoneal Mφ from wild-type, *Myd88* ^−/−^, *Trif* ^−/−^, and *Myd88* ^−/−^ *Trif* ^−/−^ mice were infected with *T. cruzi* for 3 h, and analyzed for *Tbx21* expression by real-time RT-PCR. Data are mean+s.d., and a representative result of at least three independent experiments. (B) RAW 264.7 cells were transiently transfected with either T-bet or NFATc1 expression vector, or both expression vectors together with the IFN-γ reporter plasmid. After 18 h of transfection, the cells were infected with *T. cruzi* for 18 h and activity of the reporter analyzed by luciferase assay. Data indicate mean+s.d., and a representative result of at least three independent experiments.

The NFAT family of transcription factors has been shown to interact with different transcription factors to effectively induce gene activation [22]. In the case of activation of the human *IFNG* promoter, T-bet (encoded by *Tbx21*) and NFAT have been shown to act synergistically on the promoter [39]. T-bet is a transcription factor that is essential for IFN-γ production in T cells and DCs [40]. *Tbx21* has also been shown to be induced by IFN-γ in monocytes and DCs [41]. In accordance with those studies, *Tbx21* was normally induced in *T. cruzi*-infected *Myd88* ^−/−^ *Trif* ^−/−^ Mφ (Figure 6A). Expression of NFATc1 alone weakly activated the *Ifng* promoter in RAW264.7 Mφ, but introduction of both NFATc1 and T-bet synergistically activated the *Ifng* promoter in RAW264.7 Mφ (Figure 6B). These findings indicate that NFATc1 mediates activation of the *Ifng* promoter together with T-bet in innate immune cells, like the case in T cells.

### Impaired *T. cruzi*-induced responses in *Nfatc1* ^−/−^ DCs

**Figure 7:**
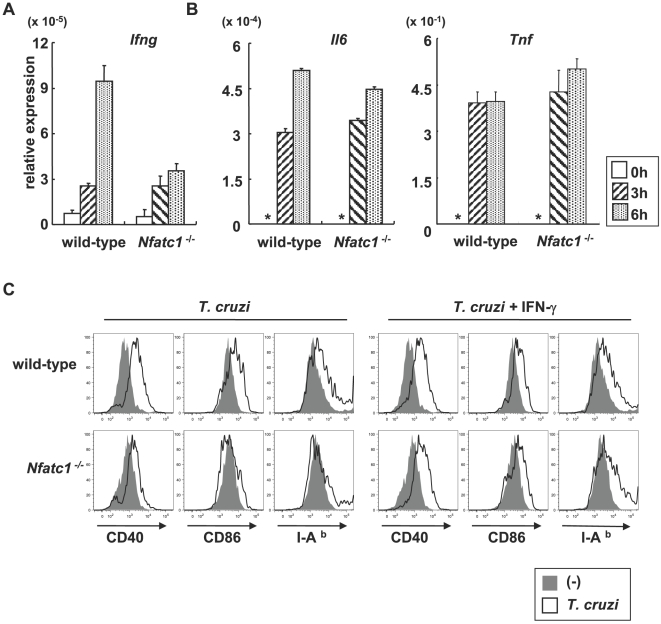
Defective *T. cruzi*-induced IFN-γ response in *Nfatc1* ^−/−^ DCs. (A, B) Wild-type and *Nfatc1* ^−/−^ FLDCs were infected with *T. cruzi* for the indicated periods. Next, total RNA was extracted, and analyzed for expression of *Ifng*, *Tnf* and *Il6*. *: not detected. (C) Wild-type and *Nfatc1* ^−/−^ FLDCs were infected with *T. cruzi* for 6 h, then washed and cultured for 24 h in the presence or absence of ρεχoμβιναντ IFN-γ. Surface expression of CD40, CD86, and I-A^b^ was analyzed by flow cytometry. Data are shown mean+s.d. of triplicate samples and a representative of four independent experiments.

To determine the role of NFATc1 in *T. cruzi*-infected DCs, we investigated IFN-γ production and maturation in *T. cruzi*-infected *Nfatc1* ^−/−^ DCs. Because mice lacking *Nfatc1* are lethal before day14.5 of gestation [42], we obtained fetal liver-derived DCs (FLDCs). Fetal liver cells of both wild-type and *Nfatc1* ^−/−^ embryos at day 12.5 of gestation differentiated into DCs expressing similar levels of CD11c in the presence of GM-CSF, Flt3 ligand and SCF (Figure S13). Moreover, LPS-induced maturation, as determined by enhanced surface expression of CD40, CD86, and MHC class II, was not impaired in *Nfatc1^−/−^* mice-derived cells (Figure S14A), indicating that development and LPS-induced maturation of *Nfatc1^−/−^* FLDCs was not compromised. *T. cruzi*-infected wild-type FLDCs expressed increased amounts of *Ifng*. In contrast, in FLDCs from *Nfatc1* ^−/−^ embryos the *T. cruzi*-induced *Ifng* expression was impaired (Figure 7A). On the other hand, *T. cruzi*-induced *Il6* and *Tnf* expression was observed normally in *Nfatc1* ^−/−^ FLDCs (Figure 7B). In *T. cruzi*-infected *Nfatc1* ^−/−^ FLDCs, expression of *Il12b* (encoding IL-12p40) was partially decreased (Figure S14B). Next, we analyzed *T. cruzi*-induced expression of MHC class II and co-stimulatory molecules on wild-type and *Nfatc1* ^−/−^ FLDCs. In *Nfatc1* ^−/−^ FLDCs, *T. cruzi*-mediated enhancement of CD40, CD86, and MHC class II was dramatically reduced (Figure 7C). The impaired surface expression of these molecules in *Nfatc1^−/−^* FLDCs was rescued by addition of exogenous IFN-γ (Figure 7C). These results indicate that NFATc1 mediates *T. cruzi*-induced IFN-γ production and maturation of DCs.

## Discussion

In the present study, we analyzed TLR-independent innate immune responses against the intracellular protozoan parasite *T. cruzi*. *T. cruzi*-infected *Myd88* ^−/−^ *Trif* ^−/−^ mice displayed normal Th1 responses and normal DC maturation. A comprehensive analysis of gene expression profiles of *T. cruzi*-infected DCs identified IFN-γ as a TLR-independent gene which mediated DC maturation and Th1 responses even in the absence of TLR signaling. *T. cruzi* infection induced an increase in intracellular Ca^2+^ level in DCs and macrophages, which led to NFATc1 activation and IFN-γ induction in a TLR-independent manner. In *Nfatc1* ^−/−^ DCs, *T. cruzi*-induced IFN-γ production and DC maturation was impaired. These findings demonstrate that NFATc1 is responsible for TLR-independent innate immune responses during *T. cruzi* infection.

The family of TLRs has been established to be critical for the innate recognition of *T. cruzi* [14]. TLR signaling pathways consist of two major components mediated by MyD88 and TRIF [43]. *Myd88* ^−/−^ mice show a high susceptibility to *T. cruzi* infection [15],[16], while mice deficient in both MyD88 and TRIF are even more susceptible to *T. cruzi* infection [17]. These findings indicate that TLR-dependent recognition of *T. cruzi* is crucial to the host defense against the parasite. In this regard, TLR-dependent induction of IFN-β might be responsible for high susceptibility to *T. cruzi* infection in *Myd88* ^−/−^ *Trif* ^−/−^ mice in spite of the normal Th1 responses [17].

A previous study showed the MyD88-dependent IFN-γ production in *T. cruzi*-infected mice [16]. However, surprisingly, we found that *Myd88^−/−^Trif^−/−^* mice exhibited normal Th1-dependent IFN-γ production. Discrepancy between both studies might be due to distinct experimental protocols. IFN-γ is known to facilitate IL-12 production. Indeed, IL-12p40-deficient mice were highly susceptible to *T. cruzi* infection with severely reduced Th1 responses [33],[34]. In *T. cruzi*-infected *Myd88* ^−/−^ *Trif* ^−/−^ mice, IL-12p40 production was severely reduced, but still induced [17], suggesting that IL-12 is produced via TLR-dependent and -independent pathways. Considering that *T. cruzi*-infected *Ifngr1^−/−^* mice showed decreased level of serum IL-12p40 and that *T. cruzi*-infected *Nfatc1^−/−^* FLDCs exhibited reduced expression of IL-12p40, NFATc1-dependent IFN-γ production may facilitate IL-12p40 production. Alternatively, the direct involvement of NFATc1 in activation of IL-12p40 gene has been also shown [23]. Collectively, *T. cruzi* infection might cause not only the TLR-dependent IL-12p40 production, but also the NFATc1-mediated (TLR-independent) production of IFN-γ and IL-12p40, coordinating the host Th1 response.

IFN-γ was identified as a gene induced in *T. cruzi*-infected *Myd88* ^−/−^ *Trif* ^−/−^ DCs. IFN-γ production by DCs was first demonstrated in IL-12-stimulated CD8α^+^ lymphoid DCs [44]. Subsequently, CD11c^low^B220^+^NK1.1^+^ cells were shown to produce high amounts of IFN-γ in response to IL-12 or a TLR9 ligand [45],[46]. These CD11c^low^B220^+^NK1.1^+^ cells have been shown to be a subset of NK cells [29]–[31]. Thus, IFN-γ production from DCs as well as Mφ is controversial [47],[48]. In order to exclude the possibility that our DC or Mφ preparations were contaminated by NK cell subsets, we isolated CD11c^high^B220^−^NK1.1^−^ cells and analyzed IFN-γ production. These cells showed a severely reduced level of IL-12/IL-18-induced IFN-γ production compared with NK cells. In addition, IL-12/IL-18-induced IFN-γ expression was less than *T. cruzi*-induced expression in these cells (our unpublished data). In *Nfatc1* ^−/−^ FLDCs, IL-12/IL-18-induced IFN-γ expression was not impaired (our unpublished data). These results indicate that *T. cruzi*-induced IFN-γ production in DCs is mediated by a pathway distinct from the IL-12 (or the TLR9 ligand)-induced one in NK subsets.

Protozoan parasites including *T. cruzi* require Ca^2+^ for their survival within the host cells [19]. In addition, *T. cruzi* evokes elevation of intracellular Ca^2+^ concentration in the host cells to establish the invasion [49],[50]. Our findings indicate that *T. cruzi*-induced activation of host Ca^2+^ signaling mediates IFN-γ production. In the host cells, the family of NFAT transcription factors, which is activated by calmodulin/calcineurin, is known to bridge Ca^2+^ to promote gene expression [51]. The role of NFAT proteins has been well characterized in T lymphocytes, and can induce activation of the *Ifng* gene [22],[52]. However, the role of NFAT proteins in innate immune cells remains unclear. Several reports indicate that NFAT proteins are activated in macrophages [24],[53]. In addition, cyclosporin A, which blocks calcineurin-dependent NFAT activation, has been shown to inhibit DC functions [54],[55]. In accordance with these reports, in the present study NFATc1 was activated in *Myd88^−/−^Trif^−/−^* Mφ in response to *T. cruzi* infection. Furthermore, analysis using FLDCs derived from *Nfatc1* ^−/−^ embryos demonstrated that NFATc1 mediates *T. cruzi*-induced IFN-γ production and DC maturation. These findings establish a new signaling pathway mediating an innate immune response during *T. cruzi* infection. It is well established that the TLR-dependent pathway initiates innate immune responses against pathogens. In addition, in the case of invasion of protozoan parasites triggering activation of intracellular Ca^2+^ signaling, NFATc1 mediates the TLR-independent innate immune responses through induction of IFN-γ.

Bradykinin B_2_ receptor has been shown to mediate *T. cruzi*-dependent generation of inositol 1,4,5-trisphosphate, leading to elevated level of intracellular Ca^2+^ [56]. However, several other mechanisms that induce intracellular Ca^2+^ influx have been proposed in *T. cruzi* infection [18]. Identification of critical molecules that lead to NFATc1 activation during *T. cruzi* infection would be a future issue to be addressed. It would be also interesting in the future to analyze whether the NFAT pathway is involved in innate immune responses against other protozoan parasites such as *Toxoplasma* and *Leishmania* species.

In this study, we focused on DCs and Mφ, which initiate Th1 responses. However, in an *in vivo* condition, *T. cruzi* are expected to invade into several other types of cells than DCs and Mφ. Therefore, it is possible that the invasion of *T. cruzi* into cells of non-innate immune cell populations indirectly influences Th1 polarization *in vivo*. In the future, we should analyze whether the NFAT family of transcription factors is involved in these processes.

In summary, in the present study we revealed a new TLR-independent mechanism for the interaction between protozoan parasites and host innate immunity. Ca^2+^ is critical for both living organisms, and therefore the parasite utilizes host Ca^2+^ for its benefit. On the host side, Ca^2+^ signaling leads to activation of NFATc1 to eliminate the parasite. It would be interesting in the future to analyze the precise role of NFATc1 in protozoan parasite infection using innate immune cell-specific NFATc1-deficient mice.

## Materials and Methods

### Mice

All animal experiments were conducted in accordance with guidelines of the Animal Care and Use Committee of Osaka University and Kyushu University. *Myd88* ^−/−^, *Trif* ^−/−^, *Ifngr1* ^−/−^, and *Nfatc1* ^−/−^ mice were generated as previously described [17],[42]. Each mouse strain was backcrossed to C57BL/6 for at least five generations, and then used to generate double or triple-mutant mice.

### Reagents

PE-conjugated anti-CD11c, PE-conjugated anti IFN-γ, APC-conjugated anti-CD11c, APC-conjugated anti-CD40, FITC-conjugated anti-NK1.1, FITC-conjugated anti-CD40, FITC-conjugated anti-CD86, FITC-conjugated anti-I-A^b^ and Pacific Blue-conjugated anti-B220 antibodies were purchased from BD Pharmingen. Anti-NFATc1 and anti-β actin antibodies were purchased from Santa Cruz. Ca^2+^ ionophore A23187 (C7522), PMA (P1585), and FK506 (F4679) were purchased from Sigma. Fluo-4 AM was purchased from Invitrogen. BAPTA-AM was from Calbiochem. Cycloheximide was from Nacalai tesque.

### Preparation of macrophages (Mφ), dendritic cells (DCs), and antigen presenting cells (APC)

To isolate peritoneal Mφ, mice were i.p. injected with 2 ml of 4% thioglycolate medium (Sigma), and peritoneal exudate cells were isolated from the peritoneal cavity at three days post injection. The cells were incubated for 2 h, then washed three times with HBSS. The remaining adherent cells were used as peritoneal Mφ in experiments. To prepare bone marrow-derived DCs or Mφ, bone marrow cells were prepared from the femur and tibia, and cultured in RPMI 1640 medium supplemented with 10% fetal bovine serum (FBS), 100 µM 2-mercaptoethanol (2ME), and 10 ng/ml GM-CSF (Pepro Tech) or 30% L cell culture supernatant, respectively. After six days, the cells were used as bone marrow DCs or bone marrow Mφ in experiments. To prepare fetal liver-derived DCs (FLDCs), fetal liver (FL) were obtained from 12.5 days post-coitum murine embryos, and FL cells were dissociated by pipetting, passed through a nylon mesh, and then cultured in RPMI 1640 medium supplemented with 10% FBS, 100 µM 2ME, 20 ng/ml GM-CSF, 10 ng/ml Flt3 ligand (Pepro Tech), and 10 ng/ml SCF (Pepro Tech) as described [57]. After eight days, the floating cells were used as FLDCs in experiments. RAW 264.7 cells were transfected with the NFATc1 (pcDNA3) expression plasmid. The cells expressing NFATc1 were selected in the presence of 0.4 mg/ml G418 and cloned. Splenocytes from wild-type mice were irradiated (30 Gy) and used as APC.

### Parasite and experimental infection

The trypomastigote stage of *T. cruzi* Tulahuen strain was maintained *in vivo* in *Ifngr1* ^−/−^ mice by passages every other week or *in vitro* in LLC-MK_2_ cells by passages every four days. For *in vitro* experiments, 5×10^5^ Mφ or DCs were infected with 1.5×10^6^ trypomastigotes. For *in vivo* experiments, mice were i.p. injected with 6×10^1^ trypomastigotes or PBS. Epimastigotes of Tulahuen strain were grown at 26°C in liver infusion tryptose liquid medium, supplemented with 2.5% hemoglobin and 10% fetal calf serum.

### Real-time RT-PCR

Total RNA was isolated with TRIzol reagent (Invitrogen), and 1–2 µg of RNA was reverse transcribed using M-MLV reverse transcriptase (Promega) and random primers (Toyobo) after treatment with RQ1 DNase I (Promega). Real-time RT-PCR was performed on an ABI 7300 (Applied Biosystems) using the TaqMan Universal PCR Master Mix (Applied Biosystems). All data were normalized to the corresponding gene *Eef1a1* encoding elongation factor-1α (EF-1α) expression, and the fold difference relative to the EF-1α was shown. Amplification conditions were: 50°C (2 min), 95°C (10 min), 40 cycles of 95°C (15 s), and 60°C (60 s). Primers of *Tbx21*, *Stat1*, *Irgm*, and *Tnf* were purchased from Assay on Demand (Applied Biosystems). Sequences for EF-1α, *Il12b*, *Il6*, and *Ifng* are as follows: EF-1α probe 5′-gcacctgagcagtgaagccagctgct-3′. forward primer 5′-gcaaaaacgacccaccaatg-3′. reverse primer 5′-ggcctggatggttcaggata-3′; *Il6* probe 5′-ccttcttgggactgatgctggtgaca-3′. forward primer 5′-ctgcaagagacttccatccagtt-3′. reverse primer 5′-aagtagggaaggccgtggtt-3′; and *Ifng* probe 5′-gtcaccatccttttgccagttcctccag-3′. forward primer 5′-tcaagtggcatagatgtggaagaa-3′. reverse primer 5′-tggctctgcaggattttcatg-3′.

### Intracellular cytokine staining

Splenic cells were isolated from *T. cruzi*-infected mice at the indicated time point and stimulated with PMA and ionomycin for 4 h in the presence of 10 µg/ml brefeldin A. In experiments to detect IFN-γ production from CD11c^+^ cells, splenic cells were infected with *T. cruzi* (1∶1) for 12 h, and further cultured for 6 h in the presence of 10 µg/ml brefeldin A. After staining of surface CD11c, CD4, CD8 or NK1.1, the cells were fixed with CytopermCytofix (BD Biosciences) for 20 min and incubated with PE-conjugated anti-IFN-γ Ab. Flow cytometric analysis was performed on FACSCantoII (BD Biosciences).

### Measurement of cytokine production

For *in vivo* experiments, mice were i.p injected with *T. cruzi*, and CD4^+^ T cells were isolated from the spleen at the indicated days after the infection. 2.5×10^5^ CD4^+^ T cells were stimulated with anti-CD3 Ab or freeze-thawed *T. cruzi* in the presence of 2.5×10^5^ APC for 24 h. The culture supernatants were collected and diluted at 1∶5. ELISA was performed with anti-mouse IFN-γ Ab, avidin-HRP, and TMB solution purchased from eBioscience. Optical densities were determined at 450 nm wavelengths with reference at 570 nm. Levels of IFN- γ were calculated from the standard curve by using purified mouse IFN- γ purchased from eBioscience.

### Flow cytometry

Bone marrow DCs or FLDCs were infected with *T. cruzi* for 6 h, washed and then cultured for 24 or 48 h. The *T. cruzi*-infected cells were stained with the combination of PE-conjugated anti-CD11c and the indicated antibodies at 4°C for 20 min, and washed. Flow cytometric analysis was performed on FACSCalibur or FACSCant II flow cytometer (BD Biosciences) and using FlowJo software (Tree Star). CD11c^high^ cells and NK cells were sorted using FACS Aria (BD Biosciences). The instrumental compensation was set in each experiment using single color, 2-color or 4-color stained samples.

### Intracellular calcium measurements

This assay was performed as described [58]. In brief, bone marrow Mφ plated on glass-bottom dishes were incubated in serum-free RPMI 1640 supplemented with 2 µM Fluo-4 AM, the increase in fluorescent intensity of which indicates increased Ca^2+^ level, at 37°C for 30 min. The cells were then washed to remove the free extracellular dye, and were maintained in culture medium during the whole experiment. The analysis of changes of basal intracellular calcium concentrations in response to *T. cruzi* infection was performed using an IX71 fluorescence microscope (Olympus).

### Immunofluorescence microscope

Bone marrow Mφ were transfected with pcDNA3-NFATc1 by nucleofection (mouse macrophage nucleofector kit; Amaxa). After 24 h, the cells were infected with *T. cruzi* for 30 min, washed with Tris-buffered saline (TBS), and then fixed with 3.7% formaldehyde in TBS for 15 min at room temperature. After permeabilization with 0.2% Triton X-100, cells were washed with TBS, incubated with anti-NFATc1 antibody in TBS containing 1% bovine serum albumin, then incubated with Alexa Fluor 594-conjugated goat anti-mouse immunoglobulin G (Molecular Probes). To stain the nucleus, cells were cultured with 0.5 mg/ml 4, 6-diamidino-2-phenylindole (DAPI; Wako). Stained cells were analyzed using an LSM510 confocal microscope (CarlZeiss).

### Luciferase assay

RAW 264.7 cells were transfected with the indicated expression plasmids together with the reporter plasmid IFN-γ-Luc and the internal control plasmid phRG-TK by Nucleofection (Nucleofector Kit V; Amaxa). After 18 h, the cells were infected with *T. cruzi* for 18 h, and the luciferase activities of whole cell lysates were measured using the Dual-luciferase reporter assay system (Promega) and Lumat LD 9507 (Berthold).

### Statistical analysis

Differences between control and experimental groups were evaluated by the Student's t-test.

## Supporting Information

Figure S1 IFN-γ production from lymphocytes after *T. cruzi* infection. (A) Splenocytes were isolated from wild-type, *Myd88^−/−^* and *Myd88^−/−^Trif^−/−^* mice at 10 days after *T. cruzi* infection, and stimulated with 1 µg/ml ionomycin plus 50 ng/ml PMA. After surface staining with APC-conjugated anti-CD4 Ab, the cells were permeabilized and then stained with PE-conjugated anti-IFN-γ Ab, and analyzed by flow cytometry. Representative results are shown from four independent experiments. The percentages of IFN-γ-producing CD4^+^ cells of individual mice are shown. (B) Splenocytes were isolated from wild-type and *Myd88^−/−^Trif^−/−^* mice at 10 days after *T. cruzi* infection, and stimulated with 1 µg/ml ionomycin plus 50 ng/ml PMA. After surface staining with FITC-conjugated anti-NK1.1 and CD8 Ab, cells were permeabilized and then stained with PE-conjugated anti-IFN-γ Ab, and analyzed by flow cytometry. Representative results are shown from two independent experiments. The percentages of IFN-γ-producing NK1.1^+^ or CD8^+^ cells of individual mice are shown.
(0.07 MB PDF)

Figure S2 Microarray analysis of *T. cruzi*-infected DCs. Bone marrow DCs from wild-type, *Myd88^−/−^* and *Myd88^−/−^Trif^−/−^* mice were infected with *T. cruzi* for 6 h. Then, microarray analysis was performed using 5 µg of total RNA. Data are shown in fold-increase of *T. cruzi*-infected cells compared with non-infected cells. Red colored boxes indicate genes showing defective induction. Genes shown by yellow colored boxes indicate so-called IFN-α/β-inducible genes.
(0.04 MB PDF)

Figure S3 TLR-independent expression of IFN-γ-inducible genes in *T. cruzi*-infected DCs. Bone marrow DCs from wild-type, *Myd88^−/−^* and *Myd88^−/−^Trif^−/−^* mice were infected with *T. cruzi* for 6 h. Then, microarray analysis was performed using 5 µg of total RNA. Data are shown in fold-increase of *T. cruzi*-infected cells compared with non-infected cells. Genes shown by yellow colored boxes have been reported as IFN-γ-inducible genes.
(0.04 MB PDF)

Figure S4 Expression of IFN-γ-inducible genes in *T. cruzi*-infected peritoneal Mφ. (A, B, C) Peritoneal Mφ from wild-type, *Myd88^−/−^* , *Trif^−/−^*, *Myd88^−/−^Trif^−/−^* and *Ifngr1^−/−^* mice were infected with *T. cruzi* for the indicated periods. Total RNA was extracted, and used for real-time RT-PCR analysis using primers specific for *Ifng*, *Stat1* and *Irgm*. All data were normalized to the corresponding gene *Eef1a1* encoding elongation factor-1α (EF1α) expression, and the fold difference relative to the EF1α was shown. Data are a representative of three independent experiments. *; not detected.
(0.02 MB PDF)

Figure S5 Low level of IFN-γ expression in IL-12/IL-18 stimulated CD11c^high^ cells. (A) Splenic CD11c^high^ cells (CD11c^high^ B220^−^ NK1.1^−^) and NK cells (CD11c^low^ B220^+^ NK1.1^+^) were sorted by FACS Area (BD Bioscience). Numbers indicate percentages of CD11c^high^ NK1.1^−^ and CD11c^high^B220^−^ cells. (B) These cells were stimulated with 10 ng/ml IL-12 plus 10 ng/ml IL-18 for 6 h. Total RNA was isolated, and then *Ifng* mRNA expression was quantified by real-time RT-PCR and normalized to the level of EF1α. Data indicate mean+s.d. and a representative result of two independent experiments. *; not detected.
(0.03 MB PDF)

Figure S6 IFN-γ-dependent DC maturation and host defense in *T. cruzi* infection. (A) Bone marrow DCs from wild-type and *Ifngr1^−/−^* mice were stimulated with 10 ng/ml murine IFN-γ for 48 h. IFNγ-stimulated DCs were stained with the combination of PE-conjugated anti-CD11c and the indicated antibodies at 4°C for 20 min, and washed. Flow cytometric analysis was performed on FACSCanto II (BD Biosciences). (B) Wild-type (*n* = 9), *Myd88^−/−^Trif^−/−^* (*n* = 5) and *Ifngr1^−/−^* (*n* = 11) mice were intraperitoneally infected with 1×10^4^ *T. cruzi*. Serum numbers of trypomastigotes were monitored at the indicated times after infection. Note that many of *Ifngr1^−/−^* mice died before 15 days of the infection.
(0.03 MB PDF)

Figure S7 IL-12 dependent Th1 response in *T. cruzi* infection. (A) Wild-type (*n* = 4) and *Il12b^−/−^* (*n* = 4) mice were intraperitoneally infected with 60 *T. cruzi*. At 6 days after infection, CD4^+^ T cells were isolated from the spleen, and then stimulated with freeze-thawed *T. cruzi* in the presence of antigen presenting cells. After 24 h, supernatants were collected and assayed for IFN-γ production by ELISA. #:*P*<0.00066. *: not detected. (B) Wild-type (*n* = 2) and *Ifngr1^−/−^* (*n* = 2) mice were intraperitoneally infected with 60 *T. cruzi*. At 3 days postinfection with *T. cruzi*, concentrations of IL-12p40 in the sera from infected mice were quantified by ELISA. *: not detected.
(0.02 MB PDF)

Figure S8 Impaired activation of MAP kinases and NF-κB in *T. cruzi*-infected *Myd88^−/−^Trif^−/−^* innate immune cells. (A) Bone marrow DCs were infected with *T. cruzi* for the indicated periods. Cell lysates were analyzed by Western blot analysis using antibodies specifically recognizing the indicated proteins. (B) Bone marrow DCs were infected with *T. cruzi* for the indicated periods. Nuclear extracts were subjected to EMSA using a radiolabeled oligonucleotide containing the murine κB site of the TNF promoter.
(0.23 MB PDF)

Figure S9 *T. cruzi*-dependent increase on Ca^2+^ concentration in Mφ. (A) Cells showing bright fluorescence at 15 min after *T. cruzi* infection were counted. Average of numbers of cells with bright fluorescence (% in total cells counted) in twelve fields from three independent experiments (4 fields in each experiment) in ×400 magnification is shown. (B, C) Bone marrow Mφ from wild-type and *Myd88^−/−^Trif^−/−^* mice were incubated with Fluo-4AM for 30 min, then washed and infected with trypomastigotes or epimastigotes for the indicated periods. The cells were analyzed by IX71 fluorescence microscope (Olympus). Epimastigotes of Tulahuen strain were grown at 26°C in liver infusion tryptose liquid medium, supplemented with 2.5% hemoglobin and 10% fetal calf serum.
(0.23 MB PDF)

Figure S10 Effect of Ca^2+^ chelator on *T. cruzi*-induced response in Mφ. (A) Peritoneal Mφ from wild-type mice were pre-incubated with 100 µM BAPTA-AM for 30 min in the presence of Fluo-4AM, then washed and infected or none-infected with *T. cruzi* for the indicated periods. Then, cells were analyzed by fluorescence microscopy. Representative of five independent experiments. (B) Cells showing bright fluorescence were counted at 10 min after *T. cruzi* infection, and average of total fifteen fields (from five independent experiments) is shown.
(0.06 MB PDF)

Figure S11 Expression of NFAT family member in bone marrow DCs. Total RNA was isolated from bone marrow DCs of wild-type and *Myd88^−/−^Trif^−/−^*. Microarray analysis was performed from 5 µg of total RNA. Levels of mRNA expression of NFAT members are shown as average difference.
(0.01 MB PDF)

Figure S12 Ca^2+^-dependent nuclear translocation of NFATc1 in *T. cruzi*-infected Mφ. Bone marrow-derived macrophages from wild-type mice (A) and *Myd88^−/−^Trif^−/−^* mice (B) were transfected with the NFATc1 expression plasmid. Cells were treated with BAPTA-AM (100 µM) for 30 min and washed, and then infected with *T. cruzi* for 30 min. *T. cruzi*-infected cells were stained with anti-NFATc1 antibody (red) and DAPI (blue). The right panels show the percentage of nuclear translocated NFATc1. Average of number of cells with nuclear NFATc1 (% in total cells counted) in twelve fields from three independent experiments (four fields in each experiment) in ×400 magnification is shown.
(0.10 MB PDF)

Figure S13 Generation of CD11c^+^ cells from *Nfatc1^−/−^* fetal liver cells. Fetal liver cells from 12.5 d.p.c. wild-type and *Nfatc1^−/−^* embryos were cultured with 20 ng/ml GM-CSF, 10 ng/ml Flt3 ligand, and 10 ng/ml SCF for 8 days. Expression of CD11c was analyzed and shown to be comparable between both genotypes. CD11c^+^ cells were enriched by MACS (Miltenyi Biotec) and used for experiments as FLDCs.
(0.03 MB PDF)

Figure S14 Response of *Nfatc1^−/−^* FLDCs to LPS and *T. cruzi*. (A) Fetal liver DCs from 12.5 d.p.c. wild-type and *Nfatc1^−/−^* embryos were stimulated with 100 ng/ml LPS for 24 h. LPS-stimulated FLDCs were stained with the combination of PE-conjugated anti-CD11c and the indicated antibodies at 4°C for 20 min, and washed. Flow cytometric analysis was performed on FACSCalibur. (B) Fetal liver DCs from 12.5 p.d.c. wild-type and *Nfatc1^−/−^* embryos were infected with *T. cruzi* for the indicated periods. Total RNA was extracted, and used for real-time RT-PCR analysis using primers specific for *Il12b*. All data were normalized to the corresponding gene *Eef1a1* encoding elongation factor-1α (EF1α) expression, and the fold difference relative to the *Eef1α1* is shown.
(0.02 MB PDF)
