# Supplementary figures and images for: NFATc1 Mediates Toll-Like Receptor-Independent Innate Immune Responses during Trypanosoma cruzi Infection
Source: PLoS Pathog. 2009 Jul 17;5(7):e1000514. doi: 10.1371/journal.ppat.1000514 (PMC2704961; doi:10.1371/journal.ppat.1000514)

A

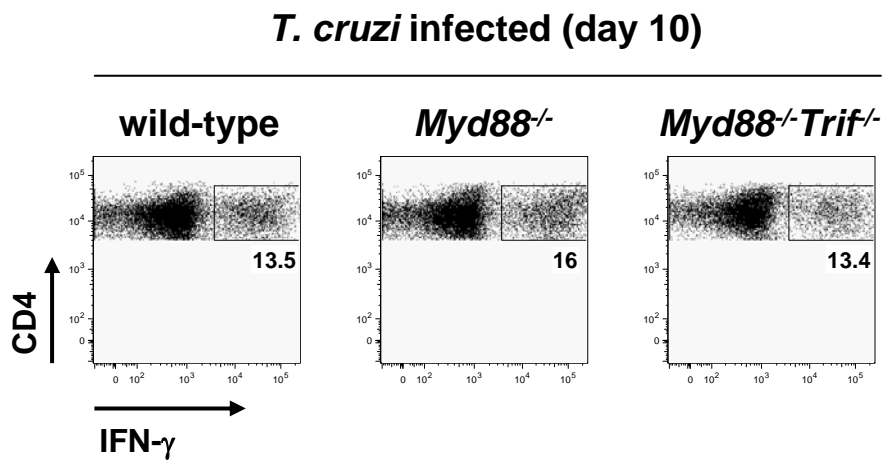

B

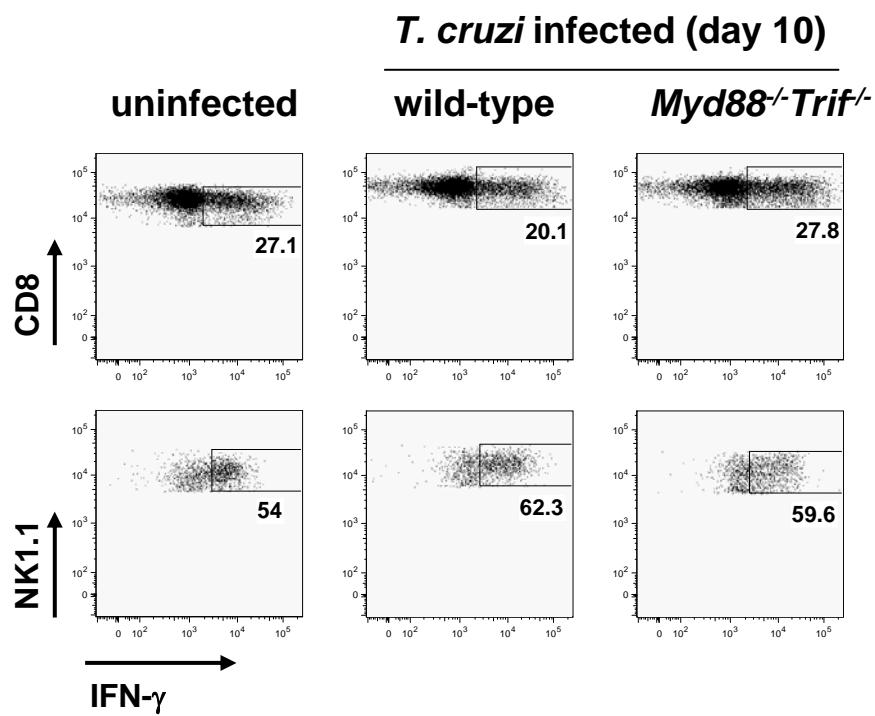

Supplement: Figure S1 — IFN-γ production from lymphocytes after T. cruzi infection. (A) Splenocytes were isolated from wild-type, Myd88−/− and Myd88−/−Trif−/− mice at 10 days after T. cruzi infection, and stimulated with 1 µg/ml ionomycin plus 50 ng/ml PMA. After surface staining with APC-conjugated anti-CD4 Ab, the cells were permeabilized and then stained with PE-conjugated anti-IFN-γ Ab, and analyzed by flow cytometry. Representative results are shown from four independent experiments. The percentages of IFN-γ-producing CD4+ cells of individual mice are shown. (B) Splenocytes were isolated from wild-type and Myd88−/−Trif−/− mice at 10 days after T. cruzi infection, and stimulated with 1 µg/ml ionomycin plus 50 ng/ml PMA. After surface staining with FITC-conjugated anti-NK1.1 and CD8 Ab, cells were permeabilized and then stained with PE-conjugated anti-IFN-γ Ab, and analyzed by flow cytometry. Representative results are shown from two independent experiments. The percentages of IFN-γ-producing NK1.1+ or CD8+ cells of individual mice are shown. (0.07 MB PDF) [file ppat.1000514.s001.pdf]

A

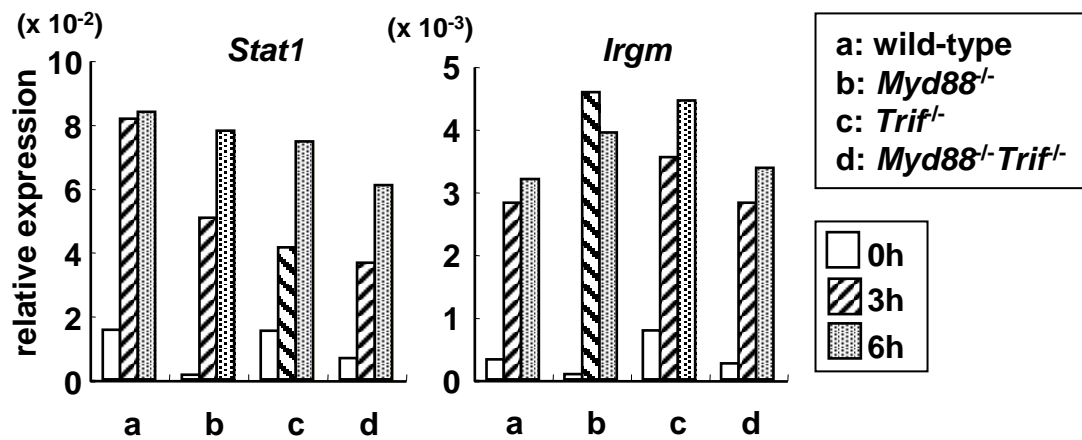

B

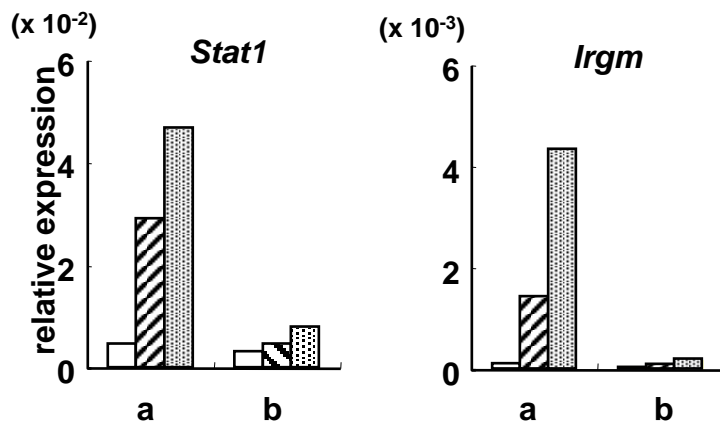

C

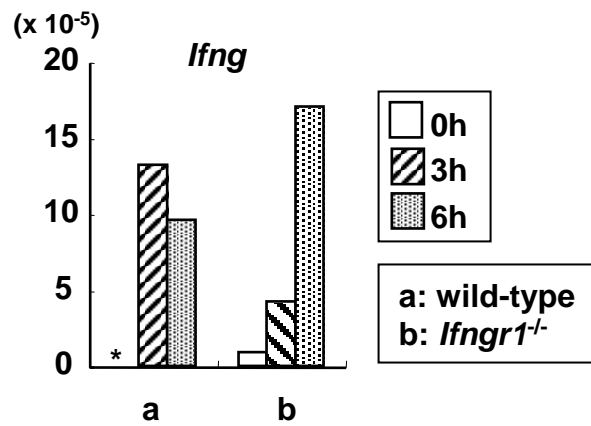

Supplement: Figure S4 — Expression of IFN-γ-inducible genes in T. cruzi-infected peritoneal Mφ. (A, B, C) Peritoneal Mφ from wild-type, Myd88−/− , Trif−/−, Myd88−/−Trif−/− and Ifngr1−/− mice were infected with T. cruzi for the indicated periods. Total RNA was extracted, and used for real-time RT-PCR analysis using primers specific for Ifng, Stat1 and Irgm. All data were normalized to the corresponding gene Eef1a1 encoding elongation factor-1α (EF1α) expression, and the fold difference relative to the EF1α was shown. Data are a representative of three independent experiments. *; not detected. (0.02 MB PDF) [file ppat.1000514.s004.pdf]

A

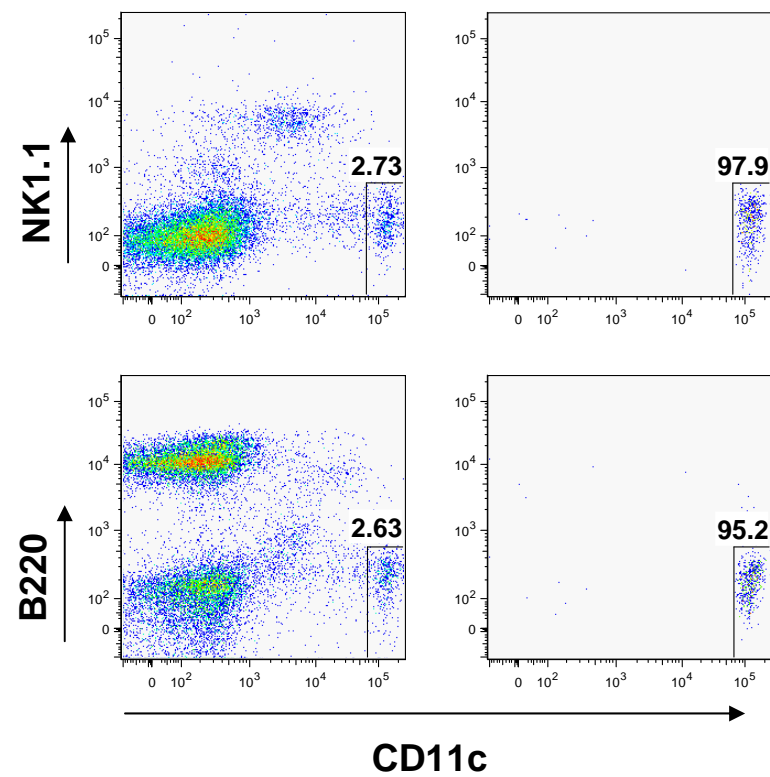

B

*Ifng*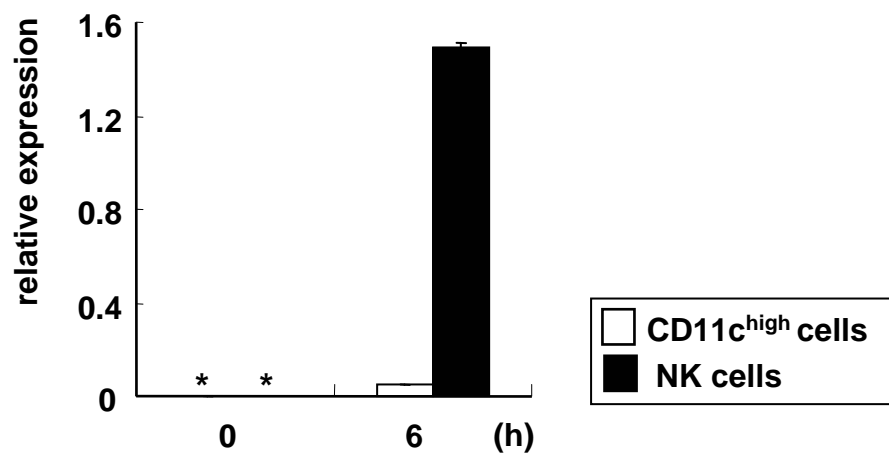

Supplement: Figure S5 — Low level of IFN-γ expression in IL-12/IL-18 stimulated CD11chigh cells. (A) Splenic CD11chigh cells (CD11chigh B220− NK1.1−) and NK cells (CD11clow B220+ NK1.1+) were sorted by FACS Area (BD Bioscience). Numbers indicate percentages of CD11chigh NK1.1− and CD11chighB220− cells. (B) These cells were stimulated with 10 ng/ml IL-12 plus 10 ng/ml IL-18 for 6 h. Total RNA was isolated, and then Ifng mRNA expression was quantified by real-time RT-PCR and normalized to the level of EF1α. Data indicate mean+s.d. and a representative result of two independent experiments. *; not detected. (0.03 MB PDF) [file ppat.1000514.s005.pdf]

A

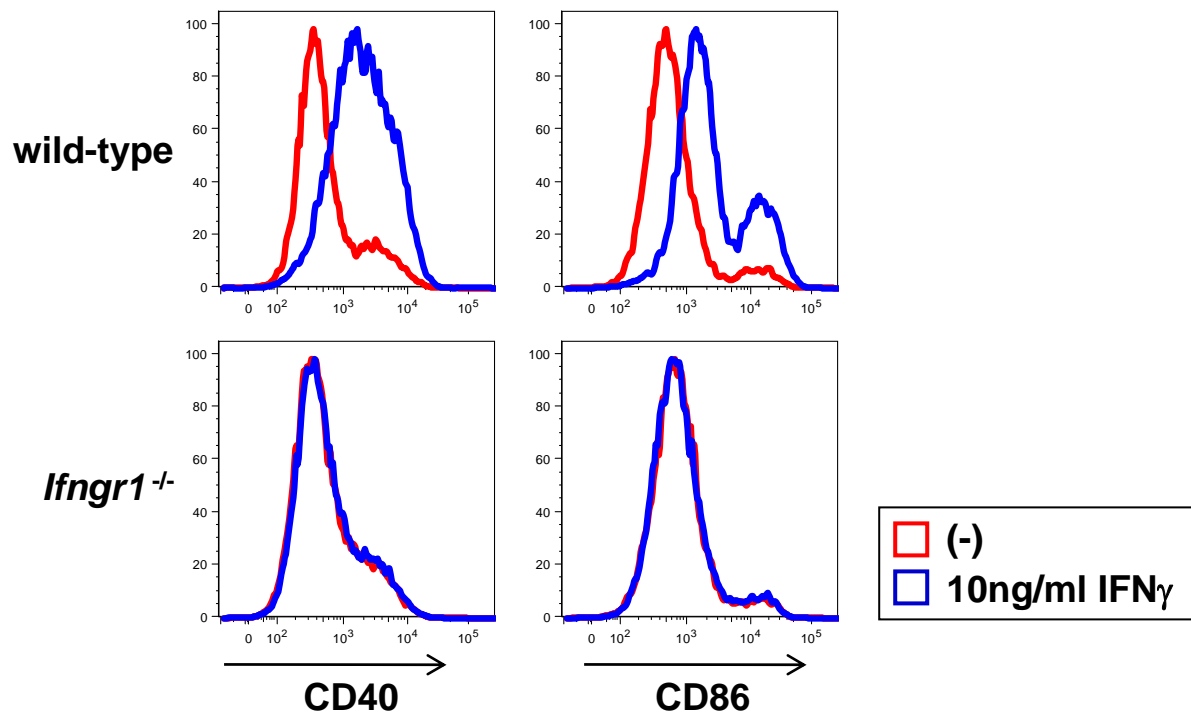

B

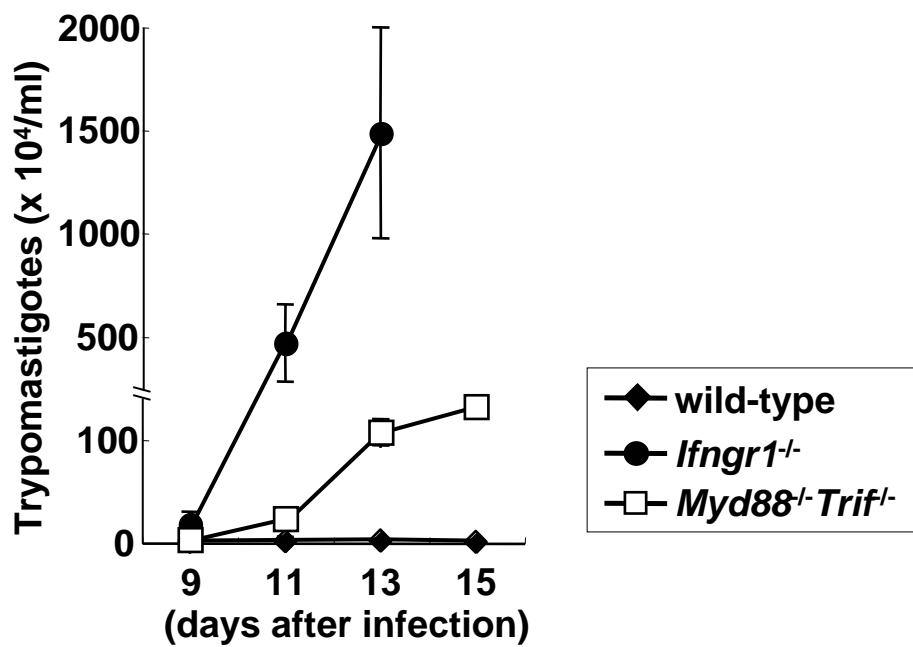

Supplement: Figure S6 — IFN-γ-dependent DC maturation and host defense in T. cruzi infection. (A) Bone marrow DCs from wild-type and Ifngr1−/− mice were stimulated with 10 ng/ml murine IFN-γ for 48 h. IFNγ-stimulated DCs were stained with the combination of PE-conjugated anti-CD11c and the indicated antibodies at 4°C for 20 min, and washed. Flow cytometric analysis was performed on FACSCanto II (BD Biosciences). (B) Wild-type (n = 9), Myd88−/−Trif−/− (n = 5) and Ifngr1−/− (n = 11) mice were intraperitoneally infected with 1×10^4^ T. cruzi. Serum numbers of trypomastigotes were monitored at the indicated times after infection. Note that many of Ifngr1−/− mice died before 15 days of the infection. (0.03 MB PDF) [file ppat.1000514.s006.pdf]

A

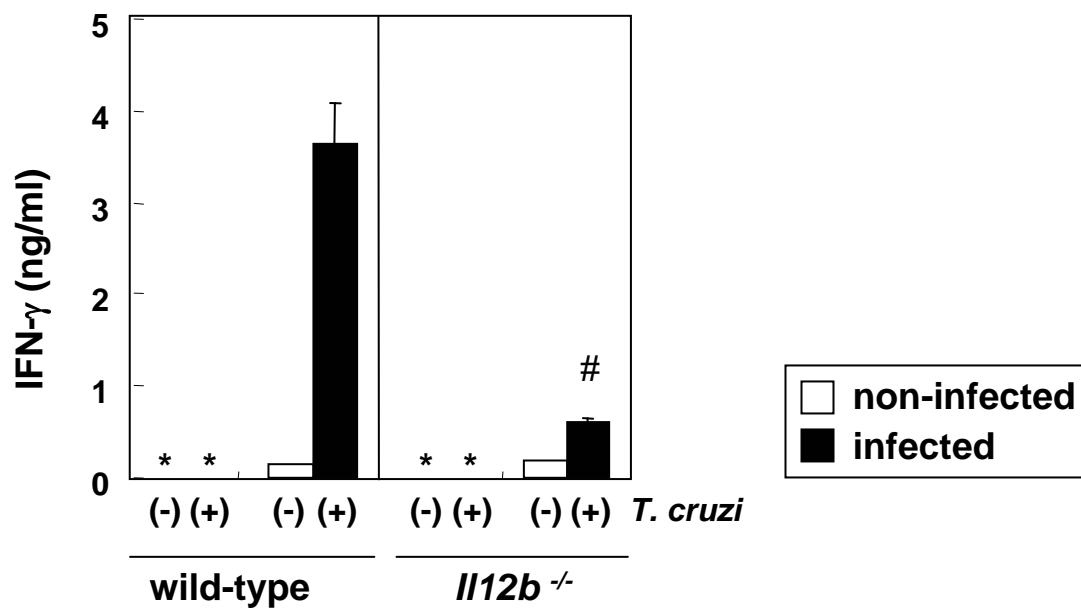

B

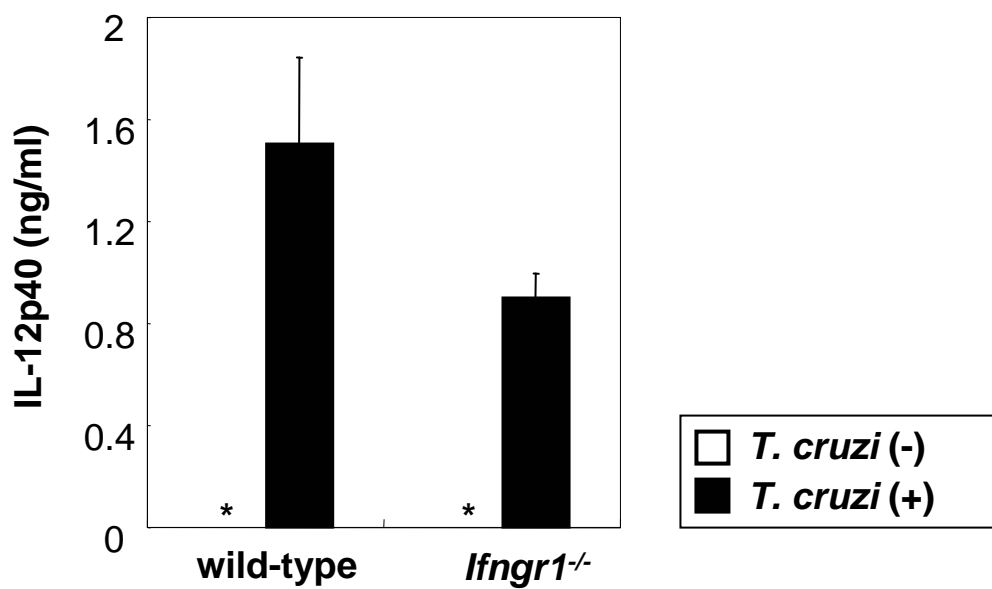

Supplement: Figure S7 — IL-12 dependent Th1 response in T. cruzi infection. (A) Wild-type (n = 4) and Il12b−/− (n = 4) mice were intraperitoneally infected with 60 T. cruzi. At 6 days after infection, CD4+ T cells were isolated from the spleen, and then stimulated with freeze-thawed T. cruzi in the presence of antigen presenting cells. After 24 h, supernatants were collected and assayed for IFN-γ production by ELISA. #:P<0.00066. *: not detected. (B) Wild-type (n = 2) and Ifngr1−/− (n = 2) mice were intraperitoneally infected with 60 T. cruzi. At 3 days postinfection with T. cruzi, concentrations of IL-12p40 in the sera from infected mice were quantified by ELISA. *: not detected. (0.02 MB PDF) [file ppat.1000514.s007.pdf]

**A**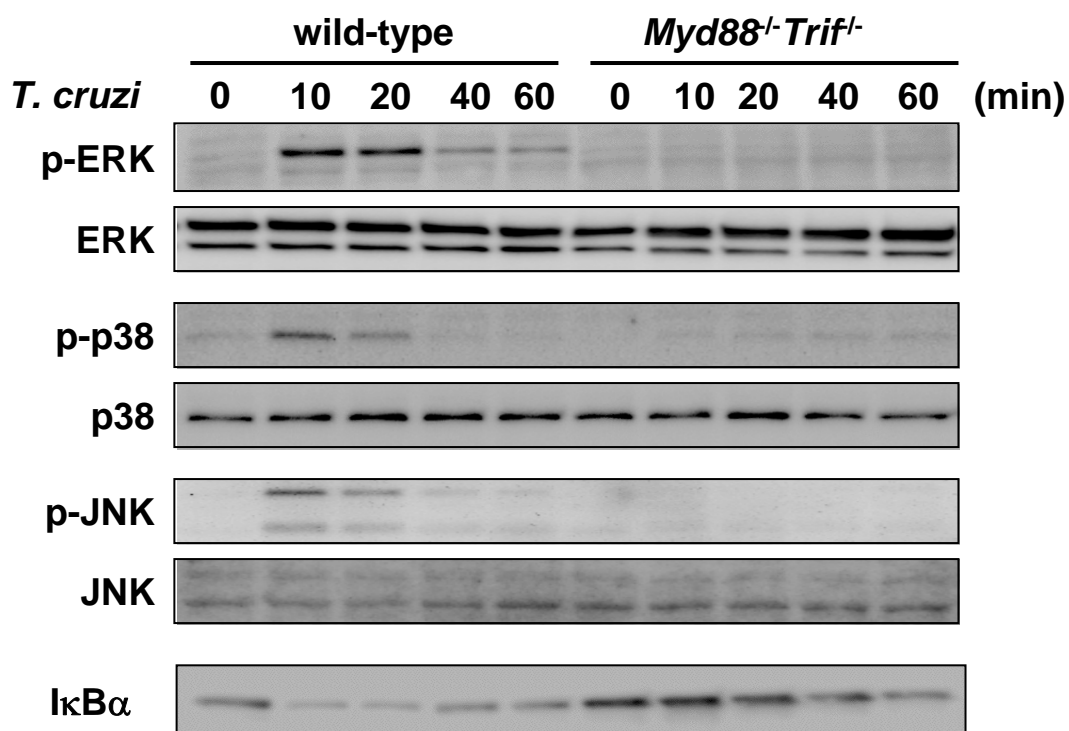**B**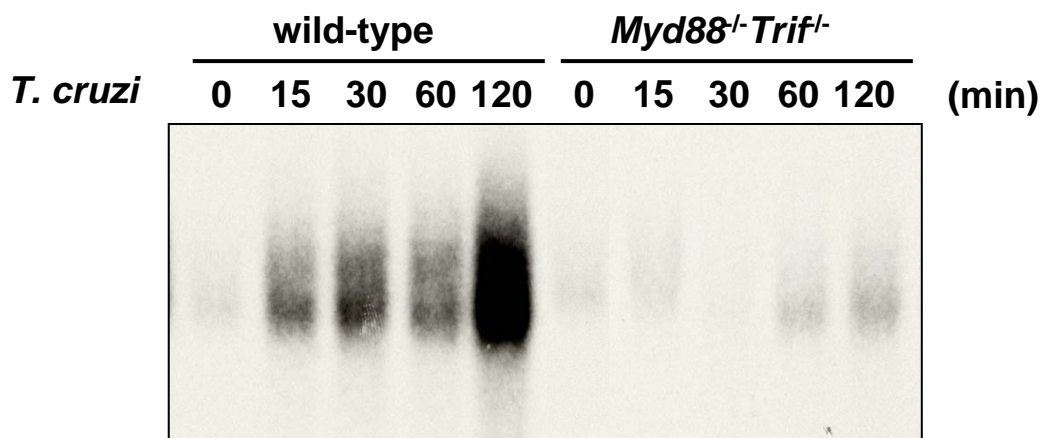

Supplement: Figure S8 — Impaired activation of MAP kinases and NF-κB in T. cruzi-infected Myd88−/−Trif−/− innate immune cells. (A) Bone marrow DCs were infected with T. cruzi for the indicated periods. Cell lysates were analyzed by Western blot analysis using antibodies specifically recognizing the indicated proteins. (B) Bone marrow DCs were infected with T. cruzi for the indicated periods. Nuclear extracts were subjected to EMSA using a radiolabeled oligonucleotide containing the murine κB site of the TNF promoter. (0.23 MB PDF) [file ppat.1000514.s008.pdf]

A

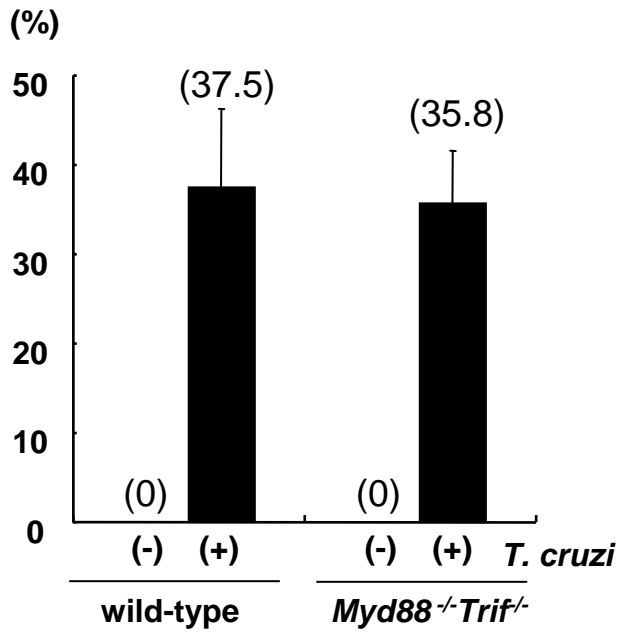

B

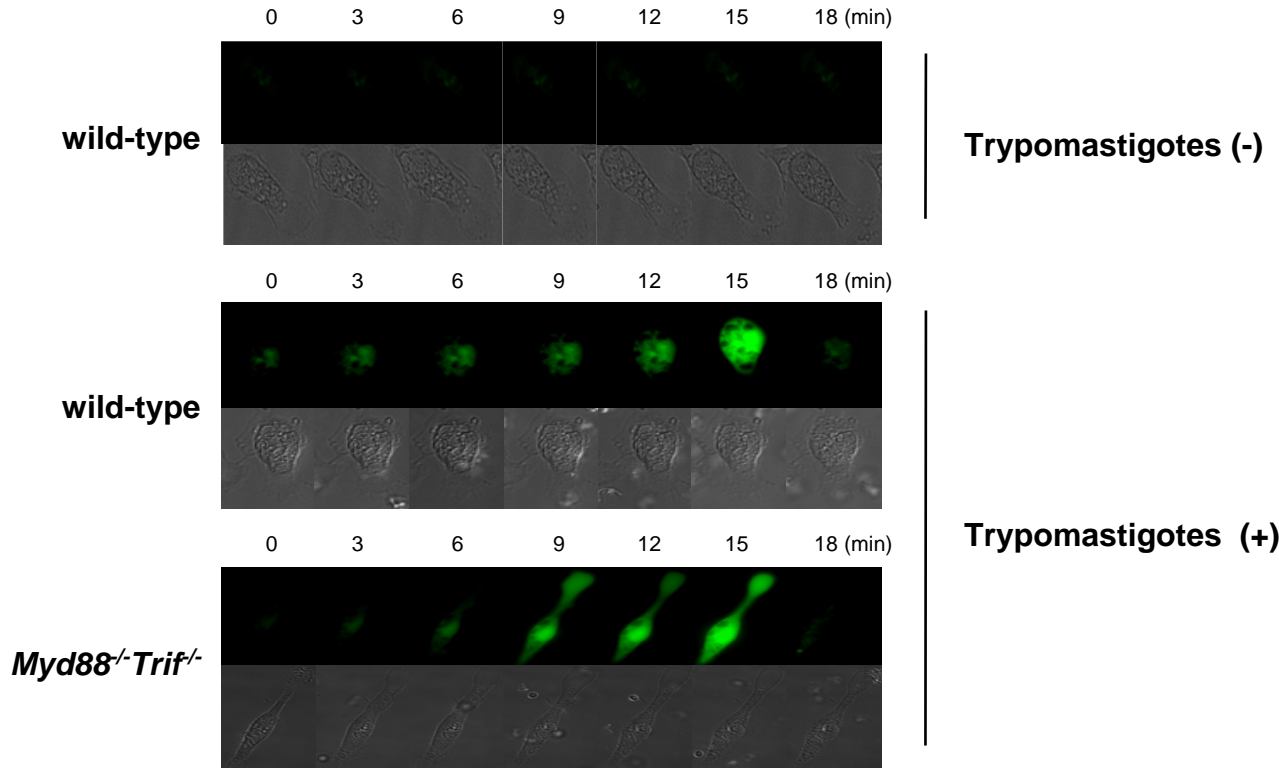

C

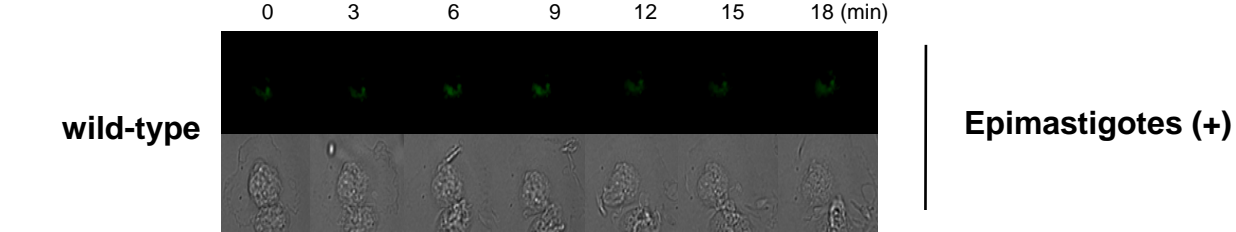

Supplement: Figure S9 — T. cruzi-dependent increase on Ca2+ concentration in Mφ. (A) Cells showing bright fluorescence at 15 min after T. cruzi infection were counted. Average of numbers of cells with bright fluorescence (% in total cells counted) in twelve fields from three independent experiments (4 fields in each experiment) in ×400 magnification is shown. (B, C) Bone marrow Mφ from wild-type and Myd88−/−Trif−/− mice were incubated with Fluo-4AM for 30 min, then washed and infected with trypomastigotes or epimastigotes for the indicated periods. The cells were analyzed by IX71 fluorescence microscope (Olympus). Epimastigotes of Tulahuen strain were grown at 26°C in liver infusion tryptose liquid medium, supplemented with 2.5% hemoglobin and 10% fetal calf serum. (0.23 MB PDF) [file ppat.1000514.s009.pdf]

A

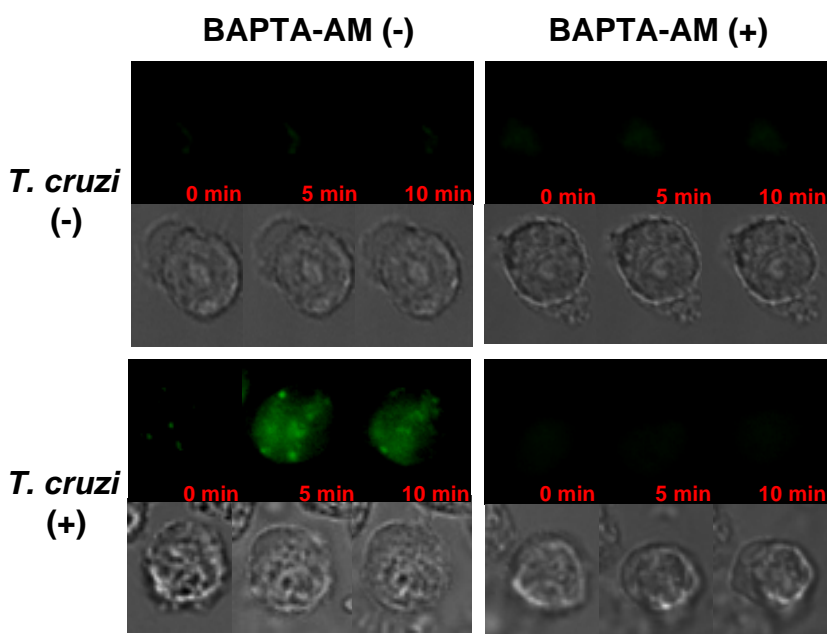

B

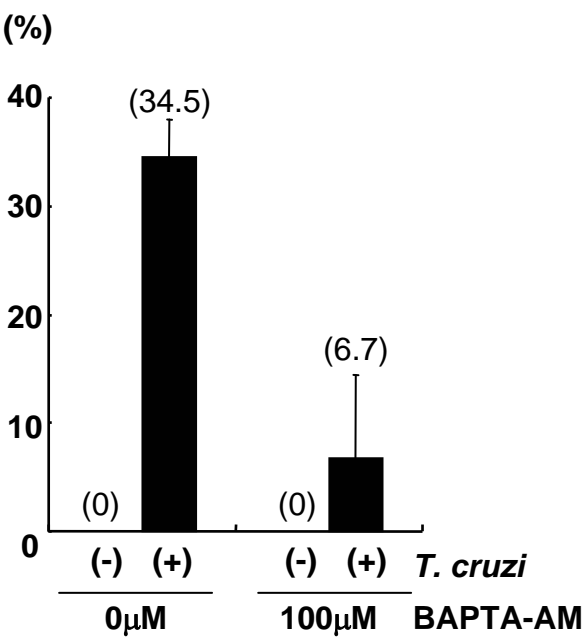

Supplement: Figure S10 — Effect of Ca2+ chelator on T. cruzi-induced response in Mφ. (A) Peritoneal Mφ from wild-type mice were pre-incubated with 100 µM BAPTA-AM for 30 min in the presence of Fluo-4AM, then washed and infected or none-infected with T. cruzi for the indicated periods. Then, cells were analyzed by fluorescence microscopy. Representative of five independent experiments. (B) Cells showing bright fluorescence were counted at 10 min after T. cruzi infection, and average of total fifteen fields (from five independent experiments) is shown. (0.06 MB PDF) [file ppat.1000514.s010.pdf]

Figure S11

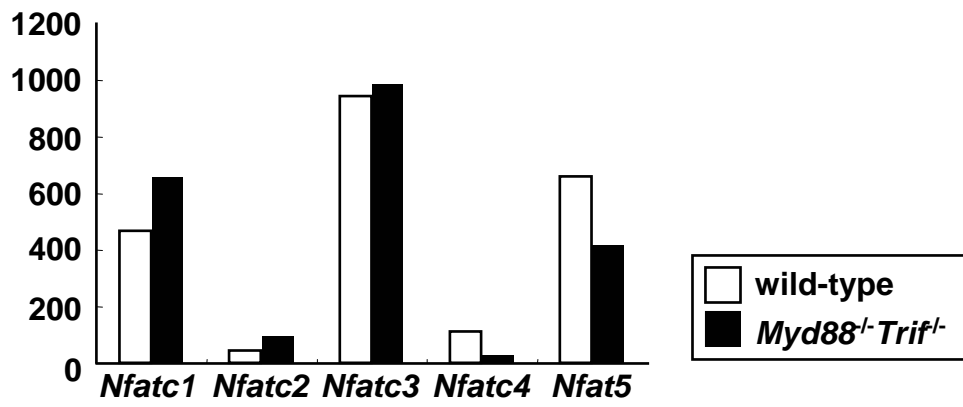

Supplement: Figure S11 — Expression of NFAT family member in bone marrow DCs. Total RNA was isolated from bone marrow DCs of wild-type and Myd88−/−Trif−/−. Microarray analysis was performed from 5 µg of total RNA. Levels of mRNA expression of NFAT members are shown as average difference. (0.01 MB PDF) [file ppat.1000514.s011.pdf]

A

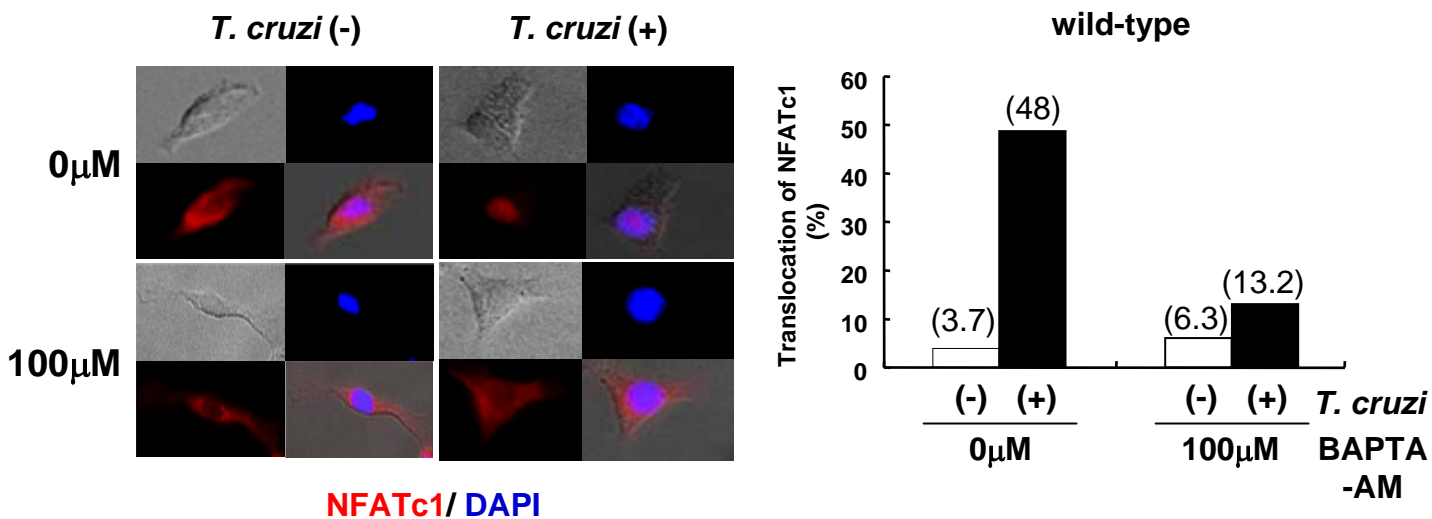

B

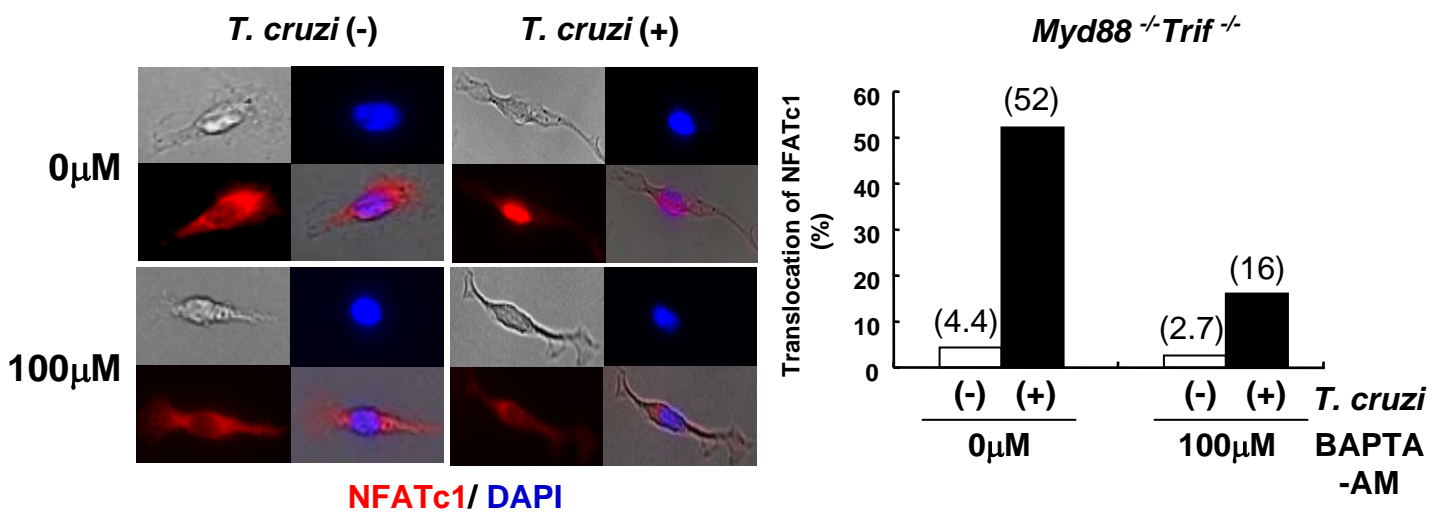

Supplement: Figure S12 — Ca2+-dependent nuclear translocation of NFATc1 in T. cruzi-infected Mφ. Bone marrow-derived macrophages from wild-type mice (A) and Myd88−/−Trif−/− mice (B) were transfected with the NFATc1 expression plasmid. Cells were treated with BAPTA-AM (100 µM) for 30 min and washed, and then infected with T. cruzi for 30 min. T. cruzi-infected cells were stained with anti-NFATc1 antibody (red) and DAPI (blue). The right panels show the percentage of nuclear translocated NFATc1. Average of number of cells with nuclear NFATc1 (% in total cells counted) in twelve fields from three independent experiments (four fields in each experiment) in ×400 magnification is shown. (0.10 MB PDF) [file ppat.1000514.s012.pdf]

Figure S13

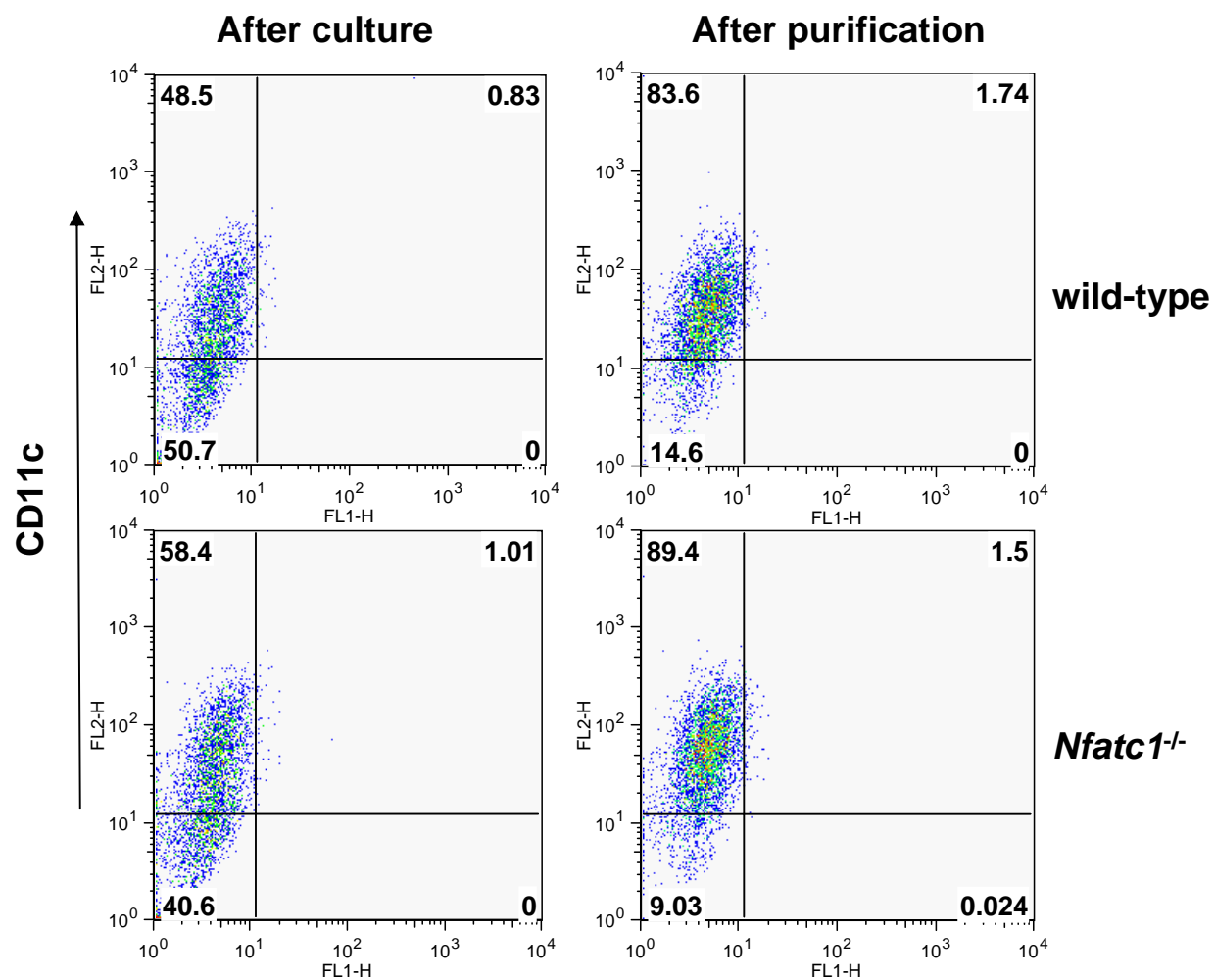

Supplement: Figure S13 — Generation of CD11c+ cells from Nfatc1−/− fetal liver cells. Fetal liver cells from 12.5 d.p.c. wild-type and Nfatc1−/− embryos were cultured with 20 ng/ml GM-CSF, 10 ng/ml Flt3 ligand, and 10 ng/ml SCF for 8 days. Expression of CD11c was analyzed and shown to be comparable between both genotypes. CD11c+ cells were enriched by MACS (Miltenyi Biotec) and used for experiments as FLDCs. (0.03 MB PDF) [file ppat.1000514.s013.pdf]

A

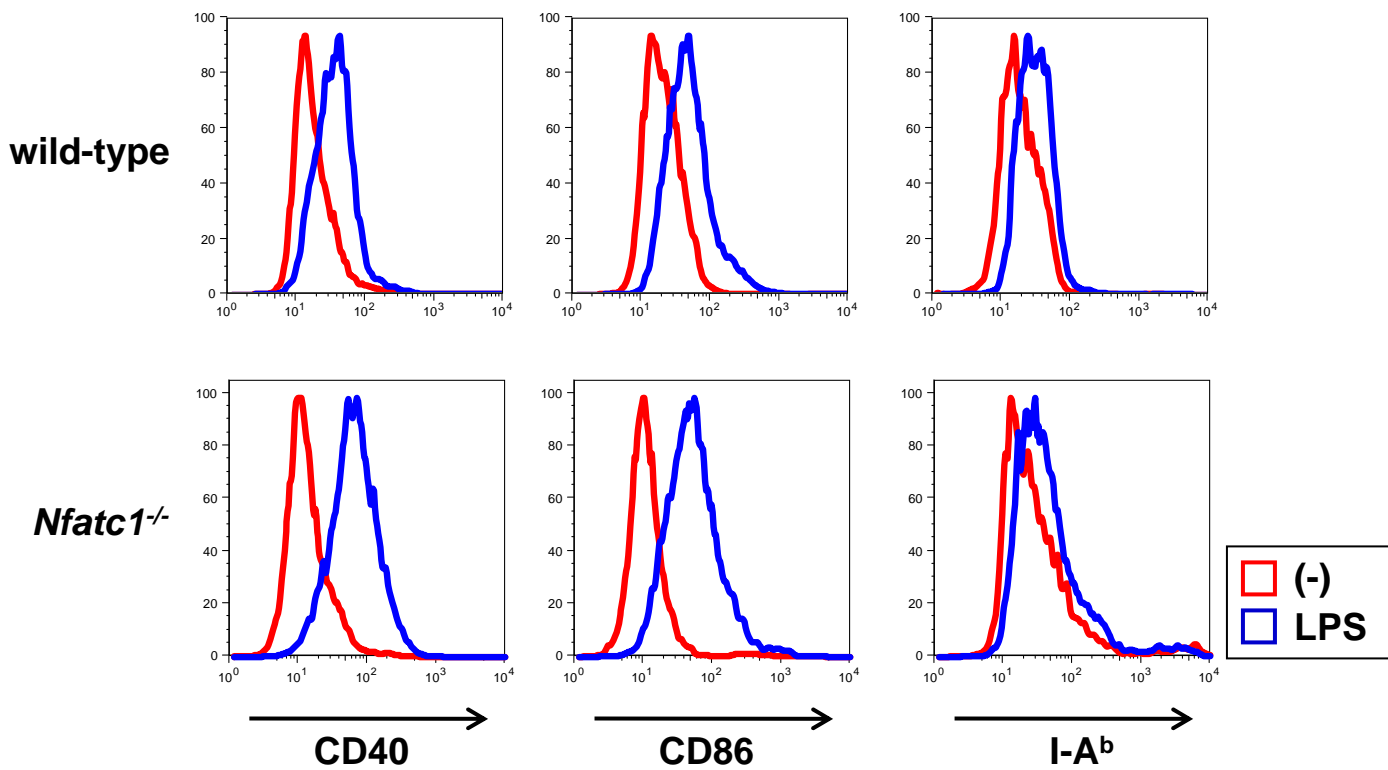

B

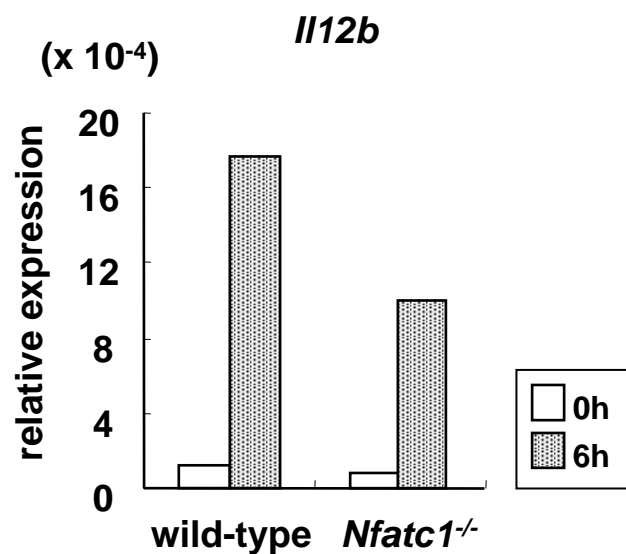

Supplement: Figure S14 — Response of Nfatc1−/− FLDCs to LPS and T. cruzi. (A) Fetal liver DCs from 12.5 d.p.c. wild-type and Nfatc1−/− embryos were stimulated with 100 ng/ml LPS for 24 h. LPS-stimulated FLDCs were stained with the combination of PE-conjugated anti-CD11c and the indicated antibodies at 4°C for 20 min, and washed. Flow cytometric analysis was performed on FACSCalibur. (B) Fetal liver DCs from 12.5 p.d.c. wild-type and Nfatc1−/− embryos were infected with T. cruzi for the indicated periods. Total RNA was extracted, and used for real-time RT-PCR analysis using primers specific for Il12b. All data were normalized to the corresponding gene Eef1a1 encoding elongation factor-1α (EF1α) expression, and the fold difference relative to the Eef1α1 is shown. (0.02 MB PDF) [file ppat.1000514.s014.pdf]
