# Supplementary material for: NFATc1 Mediates Toll-Like Receptor-Independent Innate Immune Responses during Trypanosoma cruzi Infection
Source: PLoS Pathog. 2009 Jul 17;5(7):e1000514. doi: 10.1371/journal.ppat.1000514 (PMC2704961; doi:10.1371/journal.ppat.1000514)
Supplement: Figure S2 — Microarray analysis of T. cruzi-infected DCs. Bone marrow DCs from wild-type, Myd88−/− and Myd88−/−Trif−/− mice were infected with T. cruzi for 6 h. Then, microarray analysis was performed using 5 µg of total RNA. Data are shown in fold-increase of T. cruzi-infected cells compared with non-infected cells. Red colored boxes indicate genes showing defective induction. Genes shown by yellow colored boxes indicate so-called IFN-α/β-inducible genes. (0.04 MB PDF) [file ppat.1000514.s002.pdf]

Figure S2

Section		WT(+)/(-)	MyD(+)/(-)	DKO(+)/(-)	Public ID	Gene Symbol
MyD88/TRIF-dependent genes	1419282_at	85.5	11.5	3.7	U50712	Ccl12
	1453196_a_at	44.7	11.3	0.7	BQ033138	Oasl2
	1450446_a_at	39.0	68.3	9.4	AB000710	Socs1
	1419697_at	35.9	16.5	5.2	NM_019494	Cxcl11
	1452349_x_at	34.0	11.3	1.4	AI481797	Ifi205
	1440047_at	28.7	59.5	5.9	AV237386	Socs1
	1456212_x_at	25.7	6.5	2.4	BB831725	Socs3
	1419603_at	21.5	5.8	1.2	NM_008329	Ifi16
	1416576_at	21.0	8.3	1.6	NM_007707	Socs3
	1416121_at	16.0	15.7	0.1	M65143	Lox
	1431418_at	14.8	17.0	0.9	AK021104	C030026M15Rik
	1438251_x_at	13.0	7.6	0.1	BB559067	Prss11
	1422053_at	12.2	8.3	2.6	NM_008380	Inhba
	1452348_s_at	11.9	4.7	1.4	AI481797	Ifi205
	1436555_at	11.4	4.8	1.1	AV244175	AI158848
	1425225_at	11.3	8.8	1.4	BC027310	Fcrl3
	1418761_at	10.3	4.1	0.1	BB499476	Igf2bp1
	1421228_at	10.1	3.2	0.4	AF128193	Ccl7
	1443698_at	9.4	14.7	2.6	BB645745	---
	1416625_at	9.2	3.1	0.7	NM_009776	Serping1
A piece of MyD88-dependent genes	1421473_at	49.7	1.5	1.0	BC003727	Il1a
	1419607_at	7.6	2.4	1.8	NM_013693	Tnf
	1450297_at	46.4	2.9	2.1	NM_031168	Il6
	1425454_a_at	24.8	0.1	1.1	AF128210	Il12a
	1450334_at	21.7	0.3	0.7	NM_021782	Il21
	1418803_a_at	20.8	0.9	1.4	NM_010177	Tnfsf6
	1449497_at	18.2	0.5	0.2	AF128214	Il12b
	1427624_s_at	14.2	0.2	0.5	AJ249492	Iltifb
	1450330_at	13.1	0.2	1.1	NM_010548	Il10
	1427429_at	121.2	0.7	1.9	X03019	Csf2
	1419530_at	12.6	0.5	0.3	AF128214	Il12b
	1427747_a_at	24.8	0.4	0.8	X14607	Lcn2
	1449449_at	8.5	1.9	1.3	NM_022415	Ptges
