# Supplementary material for: NFATc1 Mediates Toll-Like Receptor-Independent Innate Immune Responses during Trypanosoma cruzi Infection
Source: PLoS Pathog. 2009 Jul 17;5(7):e1000514. doi: 10.1371/journal.ppat.1000514 (PMC2704961; doi:10.1371/journal.ppat.1000514)
Supplement: Figure S3 — TLR-independent expression of IFN-γ-inducible genes in T. cruzi-infected DCs. Bone marrow DCs from wild-type, Myd88−/− and Myd88−/−Trif−/− mice were infected with T. cruzi for 6 h. Then, microarray analysis was performed using 5 µg of total RNA. Data are shown in fold-increase of T. cruzi-infected cells compared with non-infected cells. Genes shown by yellow colored boxes have been reported as IFN-γ-inducible genes. (0.04 MB PDF) [file ppat.1000514.s003.pdf]

Figure S3

MyD88/TRIF-independent genes

	WT(+)/(−)	MyD(+)/(−)	DKO(+)/(−)	Public ID	Gene Symbol
1436576_at	37.1	14.4	45.9	BB239429	A630077B13Rik
1456380_x_at	23.4	11.4	25.4	BB490338	Cnn3
1427813_at	21.9	10.3	17.9	X75636	Herc5
1425156_at	21.2	11.6	9.3	BC010229	9830147J24Rik
1435906_x_at	21.1	4.6	12.9	BE197524	Gbp2
1436836_x_at	19.5	12.8	8.1	BB724741	Cnn3
1444556_at	19.1	32.8	10.9	BB223018	Fst
1434380_at	15.2	8.2	6.7	BM241271	---
1418825_at	14.2	9.8	8.2	NM_008326	Ifi1
1436759_x_at	14.2	24.5	11.8	AV172168	Cnn3
1434458_at	14.1	23.1	22.1	BB444134	---
1417793_at	13.9	7.7	7.9	NM_019440	Igtp
1421854_at	13.5	6.3	9.4	BF136544	Fgl2
1430005_a_at	11.5	7.8	23.0	AK016990	4933430F08Rik
1455570_x_at	11.0	16.4	7.6	BB833102	Cnn3
1421365_at	10.6	10.8	7.5	NM_008046	Fst
1421855_at	10.5	4.6	4.6	BF136544	Fgl2
1450033_a_at	9.8	6.4	7.2	AW214029	Stat1
1421031_a_at	9.6	9.9	6.1	NM_023516	2310016C08Rik
1418166_at	9.6	7.5	17.6	NM_008353	Il12rb1
1430419_at	8.6	3.4	6.8	AK009549	Crsp9
1416016_at	8.4	4.0	3.0	AW048052	Tap1
1420915_at	8.0	5.8	4.4	AW214029	Stat1
